# On the history of Ludwig von Bertalanffy’s “General Systemology”, and on its relationship to cybernetics – part III: convergences and divergences

**DOI:** 10.1080/03081079.2014.1000642

**Published:** 2015-02-23

**Authors:** Manfred Drack, David Pouvreau

**Affiliations:** ^a^ Department of Theoretical Biology, University of Vienna, Vienna, Austria; ^b^ Bertalanffy Center for the Study of Systems Science, Vienna, Austria; ^c^ Department of Philosophy, University of Toulouse II, Toulouse, France

**Keywords:** general system theory, general systemology, cybernetics, Ludwig von Bertalanffy

## Abstract

Bertalanffy’s so-called “general system theory” (GST) and cybernetics were and are often confused: this calls for clarification. In this article, Bertalanffy’s conceptions and ideas are compared with those developed in cybernetics in order to investigate the differences and convergences. Bertalanffy was concerned with first order cybernetics. Nonetheless, his perspectivist epistemology is also relevant with regard to developments in second order cybernetics, and the latter is therefore also considered to some extent. W. Ross Ashby’s important role as mediator between GST and cybernetics is analysed. The respective basic epistemological approaches, scientific approaches and inherent world views are discussed. We underline the complementarity of cybernetic and “organismic” trends in systems research within the unitary hermeneutical framework of “general systemology”.

## 1. Introduction

This paper follows two earlier publications (Pouvreau and Drack 2007, Pouvreau 2014a) and marks the third and final part of this series on – what we call – “general systemology”^1^ and cybernetics. The development, scope and content of general systemology instigated by Ludwig von Bertalanffy, together with some references to cybernetics, were already outlined in part II (Pouvreau 2014a). The reader is thus referred to that part for a comprehensive insight into the basic ideas underlying the project that he termed “general system theory” (GST). The current article is based on part II. The aim here is to clarify the historical picture by investigating the conceived views of the researchers in the two intertwined fields in order to shed light – though not in a normative sense – on recent discussions and applications.^2^

The controversy between “system theorists” and “cyberneticists” already has tradition. Some of the former think of cybernetics as a mere branch of a “general system theory”, while, conversely, some of the latter see system theory as a subset of cybernetics. Others, such as Gordon Pask, did not care what name was used (Glanville 2014, 46). Accordingly, in some publications, no clear differentiation is made between cybernetics and GST (e.g. McCool 1980; cf. also Howland 1963). For Niklas Luhmann, cybernetics is one line of development within GST (Luhmann 2002, 52).^3^ In another context, it is stated that “system theorists” are often devoted to realism (cf. Dubrovsky 2004), which would contradict the constructivist approach of second order cybernetics. Ranulph Glanville – who was president of the American Society for Cybernetics (ASC) – asks: “So is there a substantive difference?”. He answers:

Cybernetics seems to be more general, more philosophical, and more abstract than [general] systems theory, which seems full of subdivisions, more pragmatic and more “real world.” Perhaps cyberneticians are fascinated by questions whereas systemists like answers. Cybernetics is also essentially concerned with dynamics: Charles François […] characterized cybernetics as the dynamic complement of systems. (Glanville 2014, 47)^4^

One may thus conclude that there are only gradual differences and no sharp borders. Claiming that GST would deal solely with structural and static models is, however, certainly not valid. To the uninformed, it might be difficult to determine what cybernetics or general systemology are, let alone differentiating between them. Part II of our series already refers to the argument of Richard Ericson, who considered it useful to distinguish between the “general systems approach” and cybernetics (Pouvreau 2014a, 190–191). This shows that there is an old but ongoing controversy within the concerned scientific community. It is not clear why general systemology and cybernetics are seen or not as one and the same thing.

The present article seeks to clarify the different and common characteristics of general systemology as Ludwig von Bertalanffy understood it, and of cybernetics. Thereby, we answer questions and correct misunderstandings that have impaired communication. We do not focus on other “system theories” that were introduced by various authors. Besides focusing on arguments made throughout Bertalanffy’s lifetime, we also pay attention to developments in cybernetics after his death in 1972. Especially, second order cybernetics and constructivism are considered. This is relevant with regard to the above-mentioned realism allegations against GST.

General systemology and cybernetics are the successors of long-back-reaching thoughts with some shared ancestors. Norbert Wiener has pointed to Gottfried Wilhelm Leibniz as the “patron saint” of cybernetics, whose philosophy focused in particular on the concepts of universal symbolism and calculus of reasoning, and who was interested in “the construction of computing machines in the mental”, containing the germs of the reasoning machine (Wiener 1948a, 12). The German mathematician and philosopher also notably influenced Bertalanffy, although with respect to other issues than those pointed out by Wiener (Pouvreau and Drack 2007, Pouvreau 2013a, 95–105, 297–302). The two projects (general systemology and cybernetics) also share younger precursors such as the proponents of Gestalt theory or Jean Piaget. von Bertalanffy (1962, 6ff) rightly emphasized that GST and cybernetics developed in parallel with different starting points (basic science versus technological applications) and different basic models (dynamic interaction and open systems versus feedback and homeostasis), but with a “communality of interest in problems of organization and teleological behavior” (von Bertalanffy 1972a, 28).

This development [a new conception of the world as organization] is indicated by the appearance of a bundle of new disciplines: *general system theory*, *cybernetics*, *information*, *decision* and *game theories*, and others. They differ in basic assumptions, models, mathematical techniques and intentions and occasionally contradict each other. They agree, however, in being “systems sciences,” concerned with aspects hitherto neglected and, in one way or the other, with problems of interaction of many variables, of organization, regulation, goal directedness and the like. (von Bertalanffy 1967, 63f; italics in the original)

Even though Bertalanffy argued otherwise, influences from one model on the other were acknowledged by other researchers such as John O. Wisdom: “The connection between open systems, steady states, and adaptation brings the subject [Bertalanffy’s “system theory”] into relation with cybernetics” (Wisdom 1951, 29). The two approaches would be appropriate to different problems: “[Certain] phenomena maybe amenable to scientific exploration by way of cybernetics, others by way of general system theory […]; or even in the same phenomenon, certain aspects may be describable in the one or the other way” (von Bertalanffy 1962, 4). But there were also attempts to combine the fields. For instance James G. Miller, with his “living systems” approach, tried to integrate aspects from cybernetics and “general system” thinking (Miller 1978; Hammond 2003, 180).

Bertalanffy had an ambivalent relationship to cybernetics and liked to stress the differences between the two approaches. Although he appreciated the great value of cybernetics for insights into regulatory, goal-seeking and teleological behaviour – which are central issues in general systemology – as well as tackling phenomena of intrinsically unknown systems, he claimed that it “falls short of being a general theory of systems, or providing a new ‘natural philosophy’” (von Bertalanffy 1967, 68). He was interested not only in the “technical” or formal features of a theory but also in its implications on other levels, such as philosophy and world view. The personal context in which Bertalanffy developed GST has to be taken into account in this regard. Details are provided in Pouvreau (2013a, 670–674, 2014a, 175–179).

An obvious convergence is evident in the fact that scientists from cybernetics conferences also joined system theorists’ conferences and vice versa. While the Society for General Systems Research (SGSR, today ISSS) was formally established in 1956, the ASC was officially established in 1964. During the 1970s, both societies became closely associated, also having common conferences (Hammond 2003). W. Ross Ashby, a key figure both in cybernetics and GST, served as the third president of the SGSR from 1962–1964 when Bertalanffy was still active. Also, later leading cyberneticists such as Stafford Beer or Heinz von Foerster served as presidents of the SGSR (Hammond 2003, 250; Pouvreau 2013a, 825–852).

Both fields comprise various dimensions, namely epistemology, scientific, application, ideology and world view. In order to compare Bertalanffy’s approach with cybernetics, it is useful to provide a short introduction into the latter field. This is given in the next section. We then compare the epistemological approaches in general systemology and cybernetics. In the next step, we investigate their basic scientific concepts. Then, characteristics of the two fields are compared with regard to world view and ideology. Finally, we examine the connections between the two fields on other levels such as the institutional level.

## 2. Origins, aims and scope of cybernetics

The system concept has far-reaching roots in history (Pouvreau 2013a, 2013b), but only in the twentieth century were many sophisticated models developed to meet the many different needs.

Broadly speaking, the modern “systems” movement has three roots. One was the demand for a “general theory of systems,” posed by myself shortly after the Second War; the second, cybernetics finding a powerful expression in Wiener’s book (1948); the third, the demands of engineering in complex production processes, man-machine systems, armament research and the like. (von Bertalanffy 1967, 61)

The concepts underlying cybernetics have a long history too – for Glanville (2014, 46), the history of cybernetics goes back to Socrates and Plato – but as a scientific field, it was developed only from the mid-twentieth century onwards. This field was initially rather technical, providing mathematical tools for the study of regulation. In this regard, it is often referred to as first order cybernetics. A few years after this endeavour started, the field was extended by applying the recursive character of regulation also to the interaction between the observed thing and the observer. Hence an epistemological approach, called second order cybernetics, emerged, which is tightly linked to concepts of first order cybernetics. Note that cyberneticists do not form a homogeneous group. Norbert Wiener and Ashby’s ideas were much closer to Bertalanffy’s thinking than those of John von Neumann and Warren McCulloch, for example.

### 2.1. First order cybernetics

The term “cybernetics” was introduced by Wiener, referring to a new field of science: “It combines under one heading the study of what in a human context is sometimes loosely described as thinking and in engineering is known as control and communication” (Wiener 1948b). This field was designed to find the “common elements” concerning the functioning of automatic machines and the nervous system, possibly leading to a theory comprising control and communication in machines and animals. The subtitle of Wiener’s influential book (1948a), in which he presents the ideas of cybernetics, provides a good impression of the scope of his approach: “Control and Communication in the Animal and the Machine”. In this book, cybernetics is defined as follows: “[The] entire field of control and communication theory, whether in the machine or in the animal” is called cybernetics by Wiener (1948a, 11).

Ashby, in turn, defined cybernetics in broader terms as the “study of systems that are open to energy but closed to information and control” – of systems that are “information-tight”^5^ (Ashby 1956, 4). Co-ordination, regulation and control were seen by Ashby as the key themes of cybernetics. Furthermore, he held that cybernetics is a “theory of machines”^6^ which is concerned not with things, but with “ways of behaving”. It is thus “essentially functional and behavioristic” (Ashby 1956, 1).

Both Wiener and Ashby were interested in using cybernetics to explain psychological phenomena, especially the malfunction of the nervous system in certain mental disorders. They thus compared failure in automata-like computing machines with psycho-pathological phenomena, whereby the focus was on functional problems compared to the structural problems occurring with lesions. Cybernetics arose in close relationship to the design of computing machines, information theory, control engineering and the study of servomechanisms, together with the anticipation of similarities between the nervous system and man-made computing machines. The neurons were seen to be analogous to relays in engineering (Wiener 1948a, 120; cf. also McCulloch and Pitts 1943). From the early days of cybernetics, the parallels between cognitive phenomena (information processing, memory, learning and regulation) and the abilities of man-made machines were at the centre of interest. Cybernetics appeared useful as a meta-disciplinary approach in various fields due to a unique combination of feedback concepts^7^ from control engineering and the mathematical theory of information introduced by Shannon and Weaver (1949).

Cybernetics may succinctly be described as the science of the regulation of systems with “circularity” or “recursiveness” as leading principles and “feedback” as a central concept.^8^ “Feedback” in those early days mostly meant *negative* feedback, where the gap between actual and reference value of a variable is decreasing. Some researchers soon stressed the necessity for cybernetics to also take into account the mechanisms of positive feedback (Wisdom 1951, 22). Here the gap is increasing.

First order cybernetics, which sought a unified explanation of brains and “purposeful” machines, was essentially grounded in two fundamental hypotheses instigated by Wiener and Ashby: (1) “that the chief mechanism of the central nervous system is one of negative feed-back”; and (2) “that the negative feed-back mechanism explains ‘purpose’ and ‘adaptive’ behavior” (Wisdom 1951, 5). Nevertheless, in the nervous system, there are also other important processes that do not involve feedback, which will be discussed below (Wisdom 1951, 22–23).

The universality of the negative feedback mechanism is illustrated by the examples of picking up a pencil or steering a boat. The act of picking up a pencil involves a feedback for the correction of the position of the fingers towards the pencil, making the distance ever smaller. A circular process from the nervous system to the muscles over “re-entering” the nervous system via the sense organs is proposed to be at work (Wiener 1948a, 6–8, 1948b). And Wiener (1948b) noted that such processes are “a step forward in the study of the nervous system as an *integrated whole*” (our italics).^9^ The steering engine which keeps a ship on course appears similar to picking up a pencil. In both cases, information about the deviation must be fed back to a control device. Subsequently, oscillation, arising due to the properties of the system, can become an issue in both cases (an example for an oscillation resulting from a brain dysfunction is cerebellar tremor or purpose tremor) (Wiener 1948a, 6–8, 96, 107; Wiener 1948b).

Key to the understanding of cybernetics is the awareness of “goal-directed” processes, “purposiveness” and “teleology”, in the living organism as well as in the machine:

The term purposeful is meant to denote that the act of behavior may be interpreted as directed to the attainment of a goal – i.e. to a final condition in which the behaving object reaches a definite correlation in time or in space with respect to another object or event. (Rosenblueth et al. 1943, 18)

**Figure 1.**
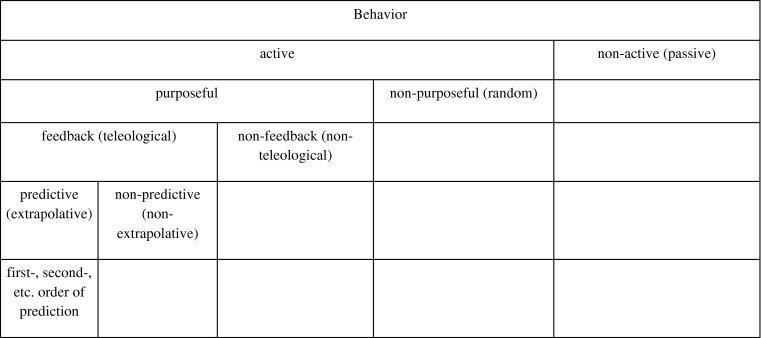
Dichotomous classification of behaviour; after Rosenblueth et al. (1943).

Purposeful active behaviour is, in the quoted article, further divided into such with feedback (teleological) and such without feedback (non-teleological).^10^ Negative feedback in such purposeful process means that an object’s behaviour “is controlled by the margin of error at which the object stands at a given time with reference to a relatively specific goal”, but therefore “some signal from the goal” is required (Rosenblueth et al. 1943, 19). Feedback purposeful behaviour can be divided further. An amoeba does not explore the path of the moving source that it follows; a behaviour that is termed non-predictive (non-extrapolative). A cat that pursues a mouse does not follow directly, but rather moves towards an extrapolated future position; a behaviour that is termed predictive (explorative). The cat predicting the path of the mouse is an instance of first-order prediction. A second-order prediction is required when throwing a stone at a moving target, where both the path of the stone and the target should be foreseen. The dichotomous classification of behaviour, as stated by Rosenblueth et al., can be depicted as in Figure 1.

Note that the term “active” here means that the output energy for a certain behavioural phenomenon stems from within the object; “in passive behavior […] all the energy in the output can be traced to the immediate input”^11^. Nevertheless, “voluntary activities” were not dismissed by Rosenblueth et al.; they rather saw them as the basis of purpose. The authors stated, in mentioning the concept of final cause, that “purposefulness, as defined here, is quite independent of causality, initial or final” (Rosenblueth et al. 1943, 23). This early text already underlines that signal, message and information were core issues in investigating goal-directed behaviour. In every feedback loop, information is passed on.

Ashby (1940) sought to define adaptiveness of animal behaviour using the concept of stable equilibrium. In his dynamic equilibrium concept, “functional circuits” (i.e. feedback loops) are essential: they enable stabilization. He mentioned examples from engineering as well as animal behaviour. The basis of his later simultaneous differential equations – where the “behavior of a given variable is […] a function of the whole system” – was already implicitly present (Ashby 1940, 481).

Ashby (1956) derived a well-developed logical basis for dealing with system transformation. By means of certain rules, one state of the system is changed to a subsequent state. This was used to model the behaviour of a dynamic system. Input from outside the “machine” can be processed internally and change the states of the system, producing changes in the output parameters. Such information-transforming “machines” can be coupled, yielding feedback loops. With respect to Bertalanffy’s GST, the changing behaviour caused by different ways of coupling is interesting:

Parts can, in general, be coupled in different ways to form a whole. The defining of the component parts does not determine the way of coupling. From this follows an important corollary. That a whole machine should be built of parts of given behavior is not sufficient to determine its behavior as a whole: only when the details of coupling are added does the whole’s behavior become determinate. (Ashby 1956, 53)

Very similar to Bertalanffy, Ashby was interested in problems in which each part of a system affects the other.^12^ He emphasized that knowing the feedback loops in a system is insufficient:

Such complex systems cannot be treated as an interlaced set of more or less independent feedback circuits, but only as a whole. For understanding the general principles of dynamic systems, therefore, the concept of feedback is inadequate in itself. What is important is that complex systems, richly cross-connected internally, have complex behaviors, and that these behaviors can be goal-seeking in complex patterns. (Ashby 1956, 54)

The concept of homeostasis as developed by Cannon (cf. 1932) influenced the early elaborations of the feedback concept. Cannon – who was influenced in that regard by Claude Bernard’s concept of the “milieu intérieur” – stressed that certain parameters are kept constant by the organism (e.g. glucose concentrations in the blood, body temperature or acid-base balance). Once a parameter deviates from an appropriate level, it is regulated towards an equilibrium (e.g. by eating when the glucose concentration is low). This already implies that a feedback mechanism might be at work. Nevertheless, there are (gradual) differences between regulations. Homeostatic feedbacks tend to be slower than others, sometimes because the involved nerve cells are non-myelinated, sometimes because the information is transmitted by hormones (Wiener 1948a, 115).

In cybernetics, the concept of a “black box” is often used, meaning something of which the internal organization is not known, and which can only be investigated by applying an input and analysing the behaviour via the output. Such a behaviouristic study procedure deals with the input and output of an object while making an abstraction of its inner organization (Rosenblueth et al. 1943). The “black box” can be a technical device as well as a living organism. With this “black box” methodology (which was in some respect congruent with Bertalanffy’s “higher order” statistical approach^13^), cybernetics broke off with some classical canons (positivist ones in particular) of scientific explanation: it studied a system by renouncing a nomothetic knowledge of its inner organization and restricting itself to the relationships between its inputs and outputs. The aim was to provide a purely phenomenological description, formulating solely qualitative hypotheses about the inner organization of the system.

The power of Wiener and Ashby’s cybernetics consisted in clearly setting the principles and aims of cybernetic modelling: to define a certain number of components and variables that are relevant for the study of a system; to unite them in a state vector; to describe the variations of this vector according to the changes of inputs or more generally the parameters external to the system; to derive the possible states of the system with regard to a definite choice of those inputs; to investigate the constraints, specific to each system, which are imposed upon the variations of the parameters; to study the specific information flux and the feedback mechanisms through which the system evolves towards a stationary state and maintains this state; to study also the potential threshold values of the characteristic variables which condition an essential change of the system’s state (Wiener 1948a; Ashby 1952b, 1956).

Besides processing of information and feedback in machines and animals, cybernetics has also played a scientific role in suggesting the relevance of such concepts in the social sciences (cf. Richardson 1991, 3). Communication in societies was considered as one of the most important factors: “the control of the means of communication is the most effective and most important” (Wiener 1948a, 160). Social systems were conceived as organizations, “bound together by a system of communication”, with dynamics in which circular processes and feedback play a major role. This was already considered in the Macy conferences – meetings organized by the Josiah Macy Jr. Foundation, starting in the 1940s and dealing with cybernetics (1946–1953)^14^. However, the application of cybernetics to sociology and anthropology, as demanded by Gregory Bateson and Margaret Mead, was an ambivalent undertaking. Wiener had doubts about progress when applying cybernetics to “present diseases of society” (Wiener 1948a, 24–25, 162):

All the great successes in precise science have been made in fields where there is a certain high degree of isolation of the phenomenon from the observer […] It is in the social sciences that the coupling between the observed phenomenon and the observer is hardest to minimize […] [W]e have to deal with short statistical runs, nor can we be sure that a considerable part of what we observe is not an artifact of our own creation […] [W]hether our investigations in the social sciences be statistical or dynamic – and they should participate in the nature of both – they can never be good to more than a very few decimal places, and, in short, can never furnish us with a quantity of verifiable, significant information which begins to compare with that which we have learned to expect in the natural sciences. (Wiener 1948a, 162–164)

Wiener, however, judged that even if cybernetics is not in position to generate mathematical theorizing in social sciences, it can bring a “conceptual clarification of the formal aspects of social relationships” (Wiener 1964, in Wittezaele and Garcia 1992, 68). It suffices here to provide the example of Karl W. Deutsch (1968) as a typical illustration for a cybernetic approach in sociology.^15^

Every application of cybernetics outside the field of engineering was confronted with a more general difficulty, not only in social sciences but also in psychology and biology. This difficulty related to the mechanistic, or at least reductionist (in the sense of the subsumption of the problems, terms and concepts of a given field under the problems, terms and concepts of another field) biases of some of its architects, or of some works usually related to its development. Those biases seriously impeded the chances of cybernetics to become a genuine transdisciplinary science, while implying ideological biases which were as pernicious as implicit (see in particular Section 5).

### 2.2. Second order cybernetics

We cannot go deeper here into the details of the development of second order cybernetics. It will be dealt with only in so far as it is necessary to understand the epistemology followed by this research direction in relation to general systemology. Although most developments of second order cybernetics occurred after Bertalanffy’s death, it is useful to compare these developments with general systemology: it enables to show how wrongly systemologists are blamed for an alleged uncritical realism (Dubrovsky 2004, 113).

Warren McCulloch, who was interested in experimental epistemology, was a predecessor of second order cyberneticists (Umpleby and Dent 1999, 94). According to Umpleby and Dent (1999, 98), cyberneticists following McCulloch’s direction were mainly concerned with cognition, adaptation and understanding, whereas “general system theorists” were mainly concerned with evolution and hierarchy, and “treated information as if it were a physical entity to be manipulated”. Wiener, with his book on *The Human Use of Human Beings*, also played a role in the first developments of second order cybernetics (Glanville 2014). Indeed, even before that book appeared, Rosenblueth and Wiener had published a paper that dealt with constructivist issues (Rosenblueth and Wiener 1945).

The concept of “cybernetics of cybernetics” was introduced by Mead (1968): she thus attempted to apply cybernetic concepts on cybernetic research itself. The term “second order cybernetics”, however, was introduced by von Foerster (cf. 1974, 1). First order cybernetics, he stated, is “[the] cybernetics of observed systems” while second order cybernetics was defined as “[the] cybernetics of observing systems”. Second order cybernetics was introduced mainly as a consequence of scientific studies of the nervous system and sense organs. Results therefrom encouraged cyberneticists to move more into the realm of philosophy of science, and such a move eventually led them to a constructivism.

The main point is that every individual constructs a personal model of the world based on his or her sensing. This need not necessarily have strict correlations to “the real world”. Moreover, the sense organs are subject to certain fallacies. Taking the optic disc or “blind spot” in the eye as an example, von Foerster (1973) liked to express that we do not see that we do not see. As we are not aware of any missing thing in our visual field, something must thus be “constructed” that is not sensed by the eye. He also noted that “perceiving is doing”, that is, sensory and motor systems are bound together by a loop in which one affects the other. Based on phenomena of the nervous system, he also rejected the idea that an individual can find out about “the reality”, but he rather saw that cognitive processes are “never ending recursive processes of computation” (von Foerster 1973). Furthermore, he pointed to the “postulate of cognitive homeostasis”: “The nervous system is organized (or organizes itself) so that it computes a stable reality” (von Foerster 1973).

Therefore, second order cyberneticists reject the attitude that observations are independent of the characteristics of the observer (Umpleby 1990).^16^ The importance of the observer is also reflected in a system definition in second order cybernetics: “A system is a set of elements and relations, or operations on these elements, that is specified by an observer. Alternatively, a system is a set of variables specified by an observer” (von Foerster 1974, 33). The set-of-variables-definition that an observer selects was already implemented by Ashby before the second order cybernetics movement gained momentum:

Because any real “machine” has an infinity of variables, from which different observers (with different aims) may reasonably make an infinity of different selections, there must first be given an observer (or experimenter); a *system* is then defined as *any set of variables* that he selects from those available on the real “machine”. It is thus a list, nominated by the observer, and is quite different in nature from the real “machine”. (Ashby 1960, 16)

Furthermore, distinguishing between organism and environment is partly conceptual and hence arbitrary (Ashby 1960, 40). As we shall see below, Ashby’s approach very much resembles that of Bertalanffy:

The point of view taken here is that science (as represented by the observer’s discoveries) is not immediately concerned with discovering what the system “really” is, but with co-ordinating the various observers’ discoveries, each of which is only a portion, or an aspect, of the whole truth. (Ashby 1956, 107)

Second order cybernetics deals with the observer and the observed as two systems interacting with each other. What this field looks at is the observer who is constructing a model of another system. In other words: “[S]econd-order cybernetics studies how observers construct models of other cybernetic systems” (Heylighen and Joslyn 2001). Or: “Second-order cybernetics in particular studies the role of the (human) observer in the construction of models of systems and other observers” (Heylighen and Joslyn 2002). Second order cybernetics thus has a meta-theoretical character.^17^

## 3. Epistemological considerations

As mentioned in the introduction, some authors detect a gap between the epistemological attitudes taken by “systemologists” and cyberneticists. It is therefore imperative to first examine the basic traits of the allegedly different approaches. We therefore provide an overview of constructivism and perspectivism, followed by considerations by the main proponents on the characteristics of scientific models.

### 3.1. Constructivism and perspectivism

Related to second order cybernetics, a (radical)^18^ constructivist philosophy of knowledge was derived. It was argued to stand in contrast to realism, specifically the realism also found in artificial intelligence approaches (Scott 2004, 1369–1370). In this regard, “realism” was conceived as the philosophy in which the world is held as primary. Theories would be only imperfect descriptions of an alleged “real” world. In the cyberneticist’s account of constructivism, the observer is meant to have more immediate access to thoughts and less to the world of experience (Umpleby and Dent 1999, 95). As mentioned above, the second order cybernetics approach to constructivism is rooted in neurophysiology. Nevertheless, constructivism already existed before certain features of the brain were discovered. What lies at the basis of cyberneticists’ constructivist epistemology was suggested by Giambattista Vico^19^ and Immanuel Kant.^20^ Jean Piaget, who developed a “genetic epistemology”, is also an important figure for constructivist cyberneticists (von Glasersfeld 1995, 147).^21^

The self-referential approach taken by second order cybernetics, where the theory has to be able to be applied to itself, stems from the concepts of self-regulation, autonomy and informational closure of cognitive organisms (cf. von Glasersfeld 1995, 149):

According to this view, reality is an interactive conception because observer and observed are a mutually dependent couple. Objectivity in the traditional sense, as Heinz von Foerster has remarked, is the cognitive version of the physiological blind spot: we do not see what we do not see. Objectivity is a subject’s delusion that observing can be done without him. Invoking objectivity is abrogating responsibility – hence its popularity. (von Glasersfeld 1995, 149)

Gaining knowledge about “reality” can be seen within the scope of negative feedback. If some concept or theory is proposed and found to be contradicted by experimental evidence, then certain characteristics of “reality” can be derived. “Reality” has for von Glasersfeld (1995, 157) the status of a black box:

The crucial aspect of our theory of knowing is that the idea of correspondence with reality is replaced by the idea of fit. Knowledge is good knowledge if it fits within the constraints of experiential reality and does not collide with them. (von Glasersfeld 1995, 156)

Whereas in the traditional view of epistemology, as well as of cognitive psychology, this relation [of knowledge and reality] is always seen as more or less picturelike (iconic) correspondence or match, radical constructivism sees it as an adaptation in the functional sense. (von Glasersfeld 1984, 20)

Coherence and invariance are important in constructivism in order to avoid complete relativism (Heylighen and Joslyn 2002). Solipsism then is not an issue. Although the validity of one model cannot be directly observed, different models from different observations lead to an increasingly coherent and reliable picture. Perceptions that do not vary much from one observation to the other, i.e. accounting for some invariants, appear to be more realistic than others. This is exactly equal to Ernst Cassirer’s and Bertalanffy’s common understanding of objectivity (Pouvreau 2013a, 322–331, 2014b, 91–95). “Another important implication of constructivism is that since all models are constructed by some observer, this observer must be included in the model for it to be complete” (Heylighen and Joslyn 2002). The effect of the observer on the observed, be it on a quantum or a social system, thus has to be taken into account.

Bertalanffy’s perspectivism and his influences (Cusanus, Kant, Spengler, Uexküll,^22^ Vaihinger, Cassirer, but also Piaget) were already addressed in the first part of our paper series (Pouvreau and Drack 2007) and in Pouvreau (2013a, [283–284, 310–313 in particular on the connection to Piaget], 2013b, 2014b). There, we showed that, according to Bertalanffy and also to Cassirer, the act of gaining knowledge is a process of objectivation; “objects” are not given to the thought, but set up by it (Pouvreau and Drack 2007, 295). In general systemology, the objects were thus not taken as “things” in themselves. The task was rather to provide the conceptual means to construct the objects’ systemicity based on “signs which are given from outside by the senses” (Pouvreau 2014a, 174).

According to Bertalanffy, the categories of knowledge depend on biological and cultural factors:

Perception is not a reflection of “real things” (whatever their metaphysical status), and knowledge not a simple approximation to “truth” or “reality.” It is an interaction between knower and known, this dependent on a multiplicity of factors of a biological, psychological, cultural, linguistic, etc., nature. […] This leads to a “perspective” philosophy […]. (von Bertalanffy 1969a, xxii)

“Absolute knowledge” is nonetheless still in a certain sense possible in the framework of this perspectivism, which thus does not fall into the trap of relativism (von Bertalanffy 1955b, 247). By looking at a “thing” from different points of view, it is, according to Bertalanffy, possible to arrive at statements about “reality”. He argued that certain aspects of “reality” are represented in the so-called “universal” physical constants (von Bertalanffy 1955b, 258–259). When taking into account different perspectives which all result in the same constants, then an objective aspect of “reality” is revealed. Cassirer first systematically related the discovery of universal constants to objective knowledge. In 1910, he identified objectivity as invariance of relationships. And in 1929, he discussed the meaning of universal constants in the here relevant regard (Cassirer 1910, 1929, 520, 552). Bernhard Bavink, who also much influenced Bertalanffy, also significantly discussed this meaning (Bavink 1949, 194–215).

Bertalanffy emphasized throughout his career the possibility of and need for searching for natural laws. Such natural laws reflect certain invariances, and science has to unveil them:

Science is interested in the invariant expressing itself in different ways. (von Bertalanffy 1953b, 236)

The only “substance” (i.e. persisting entity) left is certain invariants expressed in highly abstract conservation laws […] In its de-anthropomorphized form, science is a conceptual construct representing certain formal or structural relationships in an unknown X. (von Bertalanffy 1967, 96, 98)

Anatol Rapoport, who was here perfectly in line with Bertalanffy, made this clarifying comment:

Any structural description that reaches “all the way” to elements and relations not further analyzable may be identified with “reality” itself […] The philosophical view which declares knowledge to be knowledge of structure underlies the trend in general systems theory that puts mathematical isomorphism at the foundation. (Rapoport 1972, 48–49)

The idea that knowledge has to fit “reality”, as expressed by Glasersfeld (see above), can already be found in Bertalanffy’s considerations on the categories of experience. Bertalanffy was clearly influenced by Konrad Lorenz in this respect^23^:

[I]ts perception must allow the animal to find its way in the world. This would be impossible if the categories of experience, such as space, time, substance, causality, were entirely deceptive. The categories of experience have arisen in biological evolution, and have continually to justify themselves in the struggle for existence. If they would not, in some way, correspond to reality, appropriate reaction would be impossible, and such organism would quickly be eliminated by selection. (von Bertalanffy 1955b, 256)

It follows that there is no fundamental contradiction between Bertalanffy’s (and Cassirer’s) perspectivism and the constructivism defended by second order cyberneticists. Both can be seen as research attitudes aiming at grasping invariants in the phenomenal world via a multiplicity of conceptual constructs (“models”). Both epistemological approaches are rooted in the history of philosophy with some common ancestors, such as Vico, Kant and Piaget. There is a thematic (if not historical) continuity from general systemology to second order cybernetics. Emphasizing the observer is therefore not so much an essential extension (cf. Locker 1998). Rather, many epistemological key elements can already be found in Bertalanffy’s and his predecessors works; an example is the distinction between perception and apperception (i.e. “which features of experienced reality are focused and emphasized, and which are under-played”) (von Bertalanffy 1955b, 253–254). In second order cybernetics, the key idea is to include the observer within the domain of science (Umpleby and Dent 1999). Bertalanffy does not explicitly go as far as to include the observer into his models and the domain of science, but the observer is implicitly included in his perspectivism, which regards the model as “the essence of every knowledge in general” (von Bertalanffy 1965a, 298). Hence, even though the research priorities and emphases might be different, there is a continuity (but no contradiction) between both approaches. One potential distinction is between a “negative” approach of constructivism, in which wrong theories are proven false through application to “reality” (cf. von Glasersfeld 1984, 39), and a “positive” approach in perspectivism, where results become ever more reliable if derived from different independent starting points or perspectives. Nonetheless, the above-mentioned coherence concept of Heylighen and Joslyn would also be positive.

Perhaps the problem of realism is less one between cyberneticists and systemologists, but more one arising from different notions in applied technological versus scientific fields. Technicians aim at building things and the relevant parameters are known to them, otherwise functionality would suffer. This makes knowledge about the parts that are being dealt with a precondition. Scientists, in contrast, aim at gaining knowledge about nature, and most of them are probably aware that they cannot directly come up with knowledge about the thing itself. The engineer is aware of the inner structure and processes of the machine he is building, and thus does not demand a second order cybernetics that includes the observer (Heylighen and Joslyn 2002). Second order cyberneticists deal with organisms or social systems in which they perceive themselves as part of the observer-observed-system, and even influence the observed.

“In the classical philosophy of science, theories were presumed to have no effect on the systems they described” (Umpleby 1990). But Umpleby points out that for the case of social systems and theories about them, influences can be traced in both directions, which is seen as an “example of circular causality, self-reference, and the role of the observer”. Nonetheless, even the radical constructivists do not deny the existence of an objective reality, although von Glasersfeld (1995) prefers to speak of an “intersubjective reality”. When Dubrovsky (2004, 113) states that “[all] general system theorists share the realist view of systems”, it should anyway be underlined that this is completely false, with regard to the pioneer of general systemology and to almost all later systemologists.^24^

An even further reaching consideration is related to this emphasis on epistemological issues. The meta-theoretical character of (second order) cybernetics also finds its parallel in general systemology. Klir’s comments on such a character of “systems science”, which can be understood here as the subfield of general systemology termed “basic theoretical systemology”, are crucial in this respect:

Systems science is not a science in the ordinary sense, but rather *a new dimension in science*. Each system developed as a model of some phenomenon in any traditional science represents knowledge pertaining to that science. Knowledge in systems science is not of this kind. Rather, it is knowledge regarding knowledge structures, i.e. certain specific categories of systems [both for the model character of “systems” and for meta-theoretical self-reference]. Hence, experimental objects in systems science are not objects of the real world, but rather abstractions, which we are able to make real on the computer. (Klir 1988, 156; italics in the original)

In the framework of “basic theoretical systemology”, the systemologist is concerned with the structure of already elaborated or conceivable theoretical models of entities displaying complex behaviour; the equivalence classes are induced by the logical homologies between these models. As such, Klir’s work is clearly concerned with the model character of “systems”. But it is also concerned with self-reference insofar as he, as a systemologist, reflects upon scientific constructions in a meta-theoretical perspective.

It suffices here to show that general systemology and (second order) cybernetics do not contradict each other with respect to general epistemological issues. In some details, differences can be shown, such as the emphasis on different issues or different methods to construct knowledge: recursive loops in cybernetics and multiple perspective views in general systemology. Investigating such differences would be a useful endeavour in order to avoid talking past each other and to possibly arrive at a commonly agreed epistemological framework.

### 3.2. Awareness of the characters of models

Rosenblueth and Wiener were well aware of the specific importance and unavoidability of utilizing models in science, and they emphasized a distinction between “material” and “formal” models:

No substantial part of the universe is so simple that it can be grasped and controlled without abstraction. Abstraction consists in replacing the part of the universe under consideration by a model of similar but simpler structure. Models, formal or intellectual on the one hand, or material on the other, are thus a central necessity of scientific procedure. (Rosenblueth and Wiener 1945, 316)

A formal model is a symbolic assertion in logical terms of an idealized relatively simple situation sharing the structural properties of the original factual system. (Rosenblueth and Wiener 1945, 317)

Bertalanffy, too, was very well aware that science deals with models. The distinction between material and formal models has its analogue in von Bertalanffy’s (1965a) distinction between “substantive” and “formal” (or “structural”) models, which he made under the influence of Nagel (cf. 1961, 109–111). He saw cybernetic models of organisms or of the brain much in line with Descartes and his followers, who liked to explain the organism by machine functions (von Bertalanffy 1965a, 290). Even within general systemology, particular models are applied for specific problems (von Bertalanffy 1965a, 294). Of course, different models can be set up for different problems, but also for a single one. This view was a direct consequence of his perspectivist epistemology:

An important consideration is that the various approaches enumerated [general system theory, cybernetics and also others] are not, and should not be considered to be monopolistic. One of the important aspects of the modern changes in scientific thought is that there is no unique and all-embracing “world system.” All scientific constructions are models representing certain aspects or perspectives of reality. This even applies to theoretical physics: far from being a metaphysical presentation of ultimate reality (as the materialism of the past proclaimed and modern positivism still implies) it is but one of these models and, as recent developments show, neither exhaustive nor unique. (von Bertalanffy 1962, 4)

For certain scientific questions, models with varying degrees of appropriateness can be formulated. A homeostatic or feedback model, for instance, is appropriate for temperature regulation but not for describing growth or metabolic regulation, where the open system concept is particularly relevant (von Bertalanffy 1951a, 356–357). Bertalanffy’s distinction between GST in the “narrower sense” and GST in the “broad sense” should be emphasized and clarified in that context. (1) GST in the narrow sense refers to his considerations derived from the definition of “system” as a complex of interrelated components (that can show interaction, summative behaviour, mechanization, centralization, competition, finality, etc.). Pouvreau (2013a, 696–697) has suggested to refer to this narrow sense with the expression “organismic systemology”, which means all the aspects (theoretical as well as philosophical) of the development of a general model of the “organized system” connecting his two major “organismic” theoretical schemes of interpretation: the open system tending towards or maintaining itself in a “flux equilibrium” and “progressive hierarchization” (or “individualization”). Bertalanffy thought that this (relatively) specified systemology should be based upon a “generalized kinetics”, in a sense similar to Lotka’s use of his “fundamental equations of the kinetics of evolving systems” (Lotka 1925). (2) GST in a “broad sense” refers to a general framework mainly aiming at the development of an integrated, unified science of wholeness and organization, into which also cybernetics can be integrated (von Bertalanffy 1962, 3). GST in the “narrow sense” does not claim a monopoly (von Bertalanffy 1956a):

General systems and cybernetics are applicable to certain ranges of phenomena. In some cases either model may be applied, and the equivalence of description in the “languages” of cybernetics (feedback circuits) and dynamical system theory (interactions in a multivariable system) can be shown. We note that no scientific model is monopolistic; each may reproduce, more or less successfully, certain aspects of reality. (von Bertalanffy 1965b, 1100)

Not every model suits every problem. Exaggeration or unwarranted use of a certain model can lead to false conclusions. Bertalanffy had such concerns about models, mathematics and world view, even though he always aimed for a logico-mathematical basis for his general systemology:

Naturally, an incongruence between model and reality often exists. There are highly elaborate and sophisticated mathematical models, but it remains dubious how they can be applied to the concrete case; there are fundamental problems for which no mathematical techniques are available. Disappointment of overextended expectations has occurred. Cybernetics, e.g. proved its impact not only in technology but in basic sciences, yielding models for concrete phenomena and bringing teleological phenomena – previously tabooed – into the range of scientifically legitimate problems; but it did not yield an all-embracing explanation or grand “world view,” being an extension rather than a replacement of the mechanistic view and machine theory. (von Bertalanffy 1969a, 23)

Although Bertalanffy used differential equations in order to *illustrate* his systemology, he also found other mathematical techniques such as classical calculus, group, set, graph theory, topology and others to be fruitful when approaching systems and systems principles (von Bertalanffy 1967, 71). Bertalanffy’s systems of differential equations – which intrinsically incorporate feedback in Ashby’s sense (mentioned below, cf. also Richardson 1991, 110, 118f) – show severe limitations when confronted with certain concepts that he nonetheless also strove to include in general systemology, such as primary activity. Those equations must be seen as merely one attempt, one model within his perspectivism, relevant for the formal description of various observations.

In conclusion, the masterminds of both fields were already from the onset well aware that scientific investigation is necessarily a construction of “reality” through the lenses of provisory models.

In the present writer’s mind, GST was conceived as a working hypothesis; being a practicing scientist, he [sees] the main function of theoretical models in the explanation, prediction and control of hitherto unexplored phenomena. Others may, with equal right, emphasize the importance of [an] axiomatic approach and quote to this effect examples like the theory of probability, non-Euclidean geometries, more recently information and game theory, which were first developed as deductive mathematical fields, and later applied in physics or other sciences. There should be no quarrel about this point. The danger, in both approaches is to consider too early the theoretical model as being closed and definitive – a danger particularly important in a field like general systems which is still groping to find its correct foundations. (von Bertalanffy 1962, 6)

## 4. Scientific convergences and divergences between general systemology and cybernetics

In both general systemology and cybernetics, the main effort was to provide concepts and methods for a general science. Even though the starting points were different, the aims often overlapped. In this section, we investigate the commonalities of both scientific approaches. Since Ashby played a major role in bringing them together (cf. Scott 2004), we consider his contribution in more detail. Although the concepts often overlap, in some cases differences can be detected. Regulation and teleology are core concepts of both fields and therefore need to be discussed separately.

### 4.1. Common aim for a general science

Already in his early publications towards general systemology, Bertalanffy notes the importance of a general framework for science and thereby also refers to cybernetics:

The need for a general superstructure of science, developing principles and models that are common to different fields, has often been emphasized in recent years, for instance by the Cybernetics group of N. Wiener […]. But a clear statement of the problem and a systematic elaboration has apparently never been made. (von Bertalanffy 1950, 139)

Of course, at that time general systemology and cybernetics had just been introduced and further developments occurred. The effort of Bertalanffy’s general systemology towards the unification of science – emphasized e.g. in von Bertalanffy (1949a, 1950, 1951a, 1969a, 257–259) – seems to be more pronounced compared to the endeavours in cybernetics. His aim was to reach a meta-scientific framework by means of general systemology, which would “lead to a much-needed integration in scientific education” (von Bertalanffy 1955a). Importantly, both cybernetics and general systemology were set up to foster a general, interdisciplinary and even transdisciplinary science. Wiener and Rosenblueth, independently from Bertalanffy, shared his vision of the problems posed by the specialization of scientists, and of the importance and functions of the work in the “border fields” of established sciences. In order to develop his theses regarding general systemology in 1951, Bertalanffy partially quoted a text that Wiener had written three years earlier:

[T]he most fruitful areas for the growth of the sciences are those which have been neglected as a no-man’s land between the various established fields. Since Leibniz, […] science has been increasingly the task of specialists, in fields which show a tendency to grow progressively narrower […] *There are fields of scientific work which have been explored from the different sides of pure mathematics, statistics, electrical engineering, and neurophysiology; in which every single notion receives a separate name from each group, and in which important work has been triplicated or quadruplicated, while still other important work is delayed by the unavailability in one field of results that may have already become classical in the next field* […] Dr. Rosenblueth has always insisted that a proper exploration of these blank spaces on the map of science could only be made by a team of scientists, each a specialist in his own field but each possessing a thoroughly sound and trained acquaintance with the fields of his neighbors. (Wiener 1948a, 2–3; italics correspond to Bertalanffy’s quotation of Wiener in Bertalanffy 1951a, 337)

Hence, reasons for a general science were: taking into account the neglected “territory” between different scientific disciplines; and avoiding doing the same work time and time again. Cyberneticists and systemologists both wanted to provide a single vocabulary that would be useful to a broad variety of fields and problems. This would help to transfer knowledge from one discipline to another and thereby to gain value for the respective areas. For instance, both Wiener and Bertalanffy were interested in biological, psychological and social systems. Both approaches were much more than interdisciplinary: they both should be described as trans-disciplinary and meta-disciplinary. Ashby perfectly expressed some years later the response that cybernetics promised to offer to such demands, as well as its convergence with Bertalanffy’s aims, in characterizing it as a general science of systems called to establish and exploit “exact” isomorphisms between scientific fields:

Cybernetics offers a single vocabulary and a single set of concepts suitable for *representing the most diverse types of system* […] It offers one set of concepts that, by having *exact correspondences with each branch of science*, can thereby bring them into exact relation with one other. It has been found repeatedly in science that the discovery that two branches are related leads to each branch helping in the development of the other […] Neither, of course, can give proofs about the laws of the other, but each can give suggestions that may be of the greatest assistance and fruitfulness […] Cybernetics is likely to reveal a great number of interesting and suggestive parallelisms between machine and brain and society. And it can provide the common language by which discoveries in one branch can readily be made use of in the others. (Ashby 1956, 4; our italics)

These are practical reasons for introducing a general science. Behind these reasons lies the hypothesis that generally valid theoretical principles can be found, that have empirical relevance; this hypothesis has to be verified by further (theoretical as well as experimental) research.

As stressed by Ashby, the methodology of cybernetics enabled it to raise systemic complexity (i.e. the behaviour resistant to “meristic”^25^ analyses) to the rank of a legitimate scientific problem, and in that way to constitute itself as a *science of complex systems*:

[Cybernetics] offers a method for the scientific treatment of the system in which complexity is outstanding and too important to be ignored […] For two centuries, science has been exploring systems that are either intrinsically simple or that are capable of being analysed into simple components. The fact that such a dogma as “vary the factors one at a time” could be accepted for a century, shows that scientists were largely concerned in investigating such systems as *allowed* this method; for this method is often fundamentally impossible in the complex systems […] Until recently, science tended to evade the study of such systems, focusing its attention on those that were simple and, especially, reducible […] Science today is also taking the first steps towards studying “complexity” as a subject in its own right [… And] cybernetics offers the hope of providing effective methods for the study, and control, of systems that are intrinsically extremely complex. (Ashby 1956, 4–6)

The efficacy of cyberneticists such as Wiener and Ashby was to demonstrate the possibility to extend the realm of “exact” science to such systems by constructing mathematical theories of feedback, stability and regulation which are applicable to every phenomenon amenable to the scheme of circular causality (Wiener 1948a, chapter IV in particular; Ashby 1956). This aim of cybernetics, which makes complexity and “wholes” tangible for science above metaphysic considerations, was very much in line with Bertalanffy’s attempt in his system approach. He also meant to make graspable multivariable problems that were not tackled by the traditional sciences. Bertalanffy thus mentioned certain common features when talking about cybernetics, information theory, game theory, decision theory, topology, factor analysis and GST in the “narrower sense”.

All these theories have certain features in common. Firstly, they agree in the emphasis that something should be done about the problems characteristic of the behavioral and biological sciences, but not dealt with in conventional physical theory. Secondly, these theories introduce concepts and models novel in comparison to physics: for example, a generalized system concept, the concept of information compared to energy in physics. Thirdly, these theories are particularly concerned with multivariable problems […]. Fourthly, these models are interdisciplinary and transcend the conventional fields of science […]. Fifthly and perhaps most important: Concepts like wholeness, organization, teleology and directiveness appeared in mechanistic science to be unscientific or metaphysical. Today they are taken seriously and as amenable to scientific analysis. (von Bertalanffy 1962, 3–4)

According to Bertalanffy, both conceptions (cybernetics and GST in the “narrower sense”) aim at the same goal: finding laws of organic “wholeness” and teleology (von Bertalanffy 1951a, 353).

### 4.2. The “parallelisms” between cybernetics and general systemology as noted by Bertalanffy

Bertalanffy was well aware of Wiener’s writings. Already in the early days of both developments, he enumerated seven major “parallelisms” between GST and cybernetics (von Bertalanffy 1951a, 346, 348):

(1) The “inter-scientific character”, that is, the fact that cybernetics “embraces” different established academic disciplines. One should also note its transdisciplinarity, since Bertalanffy simultaneously pointed to the cyberneticians’ contention that “certain model conceptions are applicable in quite different fields”.
(2) The “opposition, as against the analytical, cause-effect, stimulus-response, and two-variable scheme, of the concept of mutual interaction”.^26^
(3) The “statement that there seems to correspond to the principle of complementarity in physics […] a fundamental limitation in biology, namely, a complementarity between investigation of isolated processes and that of the organism-as-a-whole”.
(4) The “emphasis laid upon the dynamic, instead of static, character of organic equilibria and the insight that the apparently solid organism is, in fact, a continuous flux” (cf. Wiener 1948a, 127).
(5) The “overcoming of the ancient antithesis of structure and function”. (This overcoming was not as clear for cyberneticists as for Bertalanffy, but the idea of the system that structures itself in functioning was present in Wiener’s conception of negative feedback: it was the ability of organisms and cybernetic machines to “produce a temporary and local reversal of the normal direction of entropy” [Wiener 1954, chapter I, II].)
(6) The “re-orientation in the concepts of time and energy”. (Bertalanffy here alluded to Wiener’s discussion (1948a, 30–44) of the significance of the transition from “Newtonian time” (reversible) to “Bergsonian time” (irreversible), where he stressed the importance of statistical conceptions).^27^
(7) The “realization that, as against the timid belief that the inductive method leads to fundamental theories and laws, the progress in physics was and in biology may be, achieved by the way of bold theoretical constructions”.

### 4.3. Bertalanffy’s interpretation of cybernetics as a specific branch of general systemology

Various opinions can be found on the relationship between general systemology and cybernetics.

Cyberneticians and systemists have always understood that there was a connection between their two fields. Some see the terms as synonyms. Often it seems the subject that is given precedence is the one in the name of the department where people studied: to systemists, cybernetics is a subset of systems, whereas to cyberneticians, systems is a limited area of cybernetics. (Glanville 2014, 46)

According to Klir, the predominant view in the systems movement is that cybernetics is a subarea of “general systems research” (Klir 2001, 49). Another expression of this view is that “from a level of abstraction of GST, cybernetics can be said to deal with theories about some special classes of systems” (Gergely 1979, 47).

From a GST perspective, the merit of cybernetics is that it explicitly introduced information flow into system approaches (von Bertalanffy et al. 1977, 146). Accordingly, cybernetics can be seen as – compared to general systemology – “solely concerned with information aspects of special as well as general systems” (Klir 1970, 162). Bertalanffy, hence, considered (first order) cybernetics as a part of his research programme. More precisely, cybernetics mainly pertains to the subfield of general systemology called “basic theoretical systemology”^28^ in Pouvreau (2013a, 2014a): it has provided sophisticated and powerful theorizing of “general systems” (such as Ashby’s “ultrastable system” model discussed below). Granted that it also has significantly contributed to the three other subfields: “applied theoretical systemology” (with the construction of relevant theoretical systemic models of specific phenomena related to regulation and control), “systems technology” (with the design of so-called “cybernetic” machines) and “philosophical systemology” (notably in connection with epistemology). The cybernetic approach is most certainly broad: it applies “the same formal structure […] to systems that are totally different with regard to their material components, the forces applied, the functions performed, and so forth” (von Bertalanffy 1967, 67). However, Bertalanffy often felt the necessity to clarify the fact that “system theory” (i.e. “organismic systemology” or GST in the “narrow sense”) and cybernetics are not the same (his argument is also based on the consideration of different kinds of regulation which are discussed in more detail below):

A clarification is required here. “System theory” is frequently identified with cybernetics, control techniques, theory of automata, and the like. It should be apparent by now that this is an error. Self-regulating mechanisms, cybernetic systems (that is, systems regulated by feedback or circular causality), and so forth, are but a rather restricted subclass of “general systems.” (von Bertalanffy 1969b, 37)

In developing in 1951 one of the first detailed criticisms of cybernetics, von Bertalanffy (1951a, 346–361) made explicit the opposition between on the one hand an “organismic” approach of systems mostly focused on the material and energetic exchanges and structured by the “open system”, “dynamical order” and “hierarchization” conceptual schemes; and on the other hand a cybernetic approach which does not deal with material and energetic exchanges and focuses on the information transfers and the feedback concept. Until 1955, Bertalanffy nonetheless never attacked cybernetics from the ethical standpoint: he focused on the scientific-philosophical and epistemological convergences and divergences between his “systemological” project and this other “science of systems” also dedicated to transdisciplinarity.

Bertalanffy’s dichotomy between fixed “structural arrangements” and “dynamic order” enabled him to deepen his reflections in that regard on the basis of his general model of “organized system”. The *theoretical* subordination (which had been constitutive of this model since the 1930s) of his second “organismic principle” (progressive hierarchization) to his first “organismic principle” (openness), was transposed by Bertalanffy at a *meta*-*theoretical* level in order to subordinate the cybernetic approach to his own (“organismic”) one. This transposition was based on the following argument:

We have to distinguish two different classes within the phenomena of regulation, finality, directiveness, and gestalt. So far as we see, the *primary* regulations are such as result from dynamic interaction within a unitary system and according to general system laws. Superimposed are those regulations which may be called *secondary*, and which are governed by fixed “machine-like” structures. They comprise all phenomena of feedback, homeostasis, and related regulative mechanisms. There is a simple reason for the primacy, in the organism, of dynamical as against structural order. In contrast to a machine which consists of lasting parts, all organic structures are maintained in a continuous process, in perpetual breaking down and regeneration. The only thing which is really permanent in the organism is the order of processes. Therefore, the ultimate reason of the pattern and order in the living system can be sought only in the laws of the process itself, not in pre-established and enduring structures. Ontogenetically as well as phylogenetically, we find a principle of progressive mechanization, creating those mechanisms and making the organism more efficient while at the same time limiting its regulability. Dynamic regulation appears to be the primordial and more general law. (von Bertalanffy 1951a, 360)

In opposition to the ideas of its founders, cybernetics could therefore not claim the status of a general science of systemic behaviour. It could not play the role of “general systemology”, its destiny being rather to become only a specific branch of the latter. The aforementioned transposition ran that way:

Feedback represents an important, but special type of system behavior. “Dynamics” is the broader theory, since we can come from general system principles, always to regulations by machines, introducing conditions of constraint, but not *vice versa*. In this sense, Cybernetics is part of a General System Theory. (von Bertalanffy 1951a, 360–361)

Importantly, those reflections, to which Bertalanffy remained faithful until the end of his life, were contemporary of the criticism of cybernetics by Wisdom (whom he very probably did not know). This criticism is, as far as we know, the first publication focused on cybernetics that mentioned Bertalanffy’s conceptions, and it is interesting that it did so by stressing both the generality and specificity of the latter’s “open system theory”, most certainly without agreeing with the idea of a reduction of cybernetics to a specific field within the framework of “general systemology” – although Wisdom knew this project:

*An interesting and important development that should be brought into relation with cybernetics is the interpretation of the organism as an open system*. This is *due to von Bertalanffy*, who has published a number of writings on system theory, and to Burton, who has briefly put forward the same idea [… Bertalanffy’s open systems theory] gives a framework into which fundamental properties of the organism fit […] Thus von Bertalanffy develops from it the concept of “equifinality” for final states, such as size attained by growth […] *System theory is not to be regarded as a part of cybernetics*, because it is a very general theory with all kinds of applications. The connection between open systems, steady states, and adaptation brings the subject into relation with cybernetics; but, in the main, system theory works on its own [… and some of its] developments, interesting as they are, are not connected with cybernetics. (Wisdom 1951, 19–20; our italics)

Although nothing suggests that these commentaries of Wisdom, published in the UK, have significantly influenced American cyberneticists, it is very probable that at least one of their English colleagues did not remain impervious to them: it is Ashby, who very early showed interest in Bertalanffy’s conceptions.^29^ The influence of Ashby’s works in the history of “general systemology” is weighty enough to merit study.

### 4.4. Ashby’s role as mediator between cybernetics and general systemology

The nature and origin of adaptive behaviour was at the heart of Ashby’s reflections as early as 1940 (Wisdom 1951, 6). According to him, this issue was closely related to what he viewed as a paradox in organic evolution: while Descartes had claimed that there must be as much reality and perfection in a cause as in its effect, the Darwinian theory of evolution claimed that one simple rule (natural selection) operating on a flux of random mutations can produce structures and an adaptation much more complex than the rule according to which they have been generated (a complexity which moreover does not depend on the rules themselves). This paradox was allegedly based on the existence of two ways of understanding what is meant by the “cause” of the state of the system: if everything that contributes to the determination of this state is included, that is, the whole set of the detailed specifications of the evolution process, then Descartes is right; but it is also possible to understand by “cause” only a part of the determination in question, and nonetheless to account in that way for the evolution process, which leads to the invalidation of the Cartesian postulate: this was Ashby’s leading hypothesis.

Ashby’s initial main concern as a psychiatrist remained brain functioning, and more particularly learning. His aim was thus to prove that it is possible to construct a machine having this ability, and to establish in that way a “machine” explanation of this ability even in humans:

I hope to show that a system can be both mechanistic in nature and yet produce behavior that is adaptive. I hope to show that the essential difference between the brain and any machine yet made is that the brain makes extensive use of a method hitherto little used in machines. I hope to show that by the use of this method a machine’s behavior may be made as adaptive as we please, and that the method may be capable of explaining even the adaptiveness of Man. (Ashby 1952b, 1)

Ashby was led by his perspective to elaborate a theory of adaptive behaviour and to construct, based on this theory, a machine meeting those aims. But in doing so, it is more generally his own conception of cybernetics that he elaborated; a conception which, while being in significant aspects different from Bertalanffy’s general systemological project, enabled an essential mediation between this project and the cybernetic science of systems.

One first important point is the radically deterministic and behaviouristic character of Ashby’s approach (Ashby 1952b, 9–11, 35). The concept of a “determinate machine” as a “dynamic system” (i.e. “that may change with time”) was what Ashby called, in describing it as an “abstraction” which “is quite different in nature from the real machine”, a “state-determined system”. The “determinate machine” is, not a “thing”, but a “list of variables” that one “selects” among the available infinity of variables in a “real machine” (the variables which are not selected in order to define this system are “parameters”). This is because these variables are judged “relevant with regard to a given interest”; their current values defining the “state” of the system and the “field” of the latter having the characteristic property that a unique “line of behavior” runs through one definite point of the associated phase space (Ashby 1952b, 13, 16–27, 71). Ashby defended this point of view as early as 1945, but also referred in 1952 to Bertalanffy’s discussion two years earlier in his “outline of general system theory”. Ashby argued that specifying the behaviour of every “determined machine” is possible if the variables are continuous, by means of a differential system whose general type is precisely the one that Lotka and Bertalanffy had considered in their respective works (Ashby 1952a, 49). However, Ashby did not use this general system in the “systemological” perspective of his colleagues. He merely discussed, according to the nature of the eigenvalues of the associated matrix, the possible types of stability when the functions are linear and, in the only case where the number of variables is limited to four, in the framework of an engineering work which we will examine further (Ashby 1952a, 54). Moreover, he did not use this differential system with the “illustrative” aim that Bertalanffy had. While he also put its generality in perspective, he meant to assert another generality, probably to take into account the many discontinuous phenomena encountered in biology or engineering: the generality of the “machine with input” (or the “finite automata”). The latter was understood by Ashby as a mapping of the set product of a set *S* of internal states and of a set *I* of inputs to the set *S* (in the form *S* × *I* → *S*). He judged this mapping “able to include both the continuous, the discontinuous and even the discrete without the slightest loss of rigor” (Ashby 1962, 261).

Despite the limitation of his real interest to “determined machines”, Ashby developed a conception of cybernetics whose convergence with Bertalanffy’s general systemological project was perfect in many respects. Nonetheless, while very “diplomatically” judging Bertalanffy’s contribution to system theorizing as “substantial and welcome”, Ashby blamed him for having too deeply rooted his reflections in problems originating in natural science, and for not having brought any contribution to the real development of a perfectly abstract and axiomatic theory:

[T]hough I entirely agree with von Bertalanffy on the desirability of a general theory of systems, I am not in sympathy with his method of proceeding […] My feeling is that a properly general theory of systems must resolutely divorce itself from actual, contemporary, physics and chemistry; it must develop first in the world of the abstract, and must come into contact with the real world only when possessed of sufficient *internal* coherence to be able to preserve its structure, which must be rich and comprehensive enough to include *any* physical system, or any real behavior, that may confront it. The position, in my opinion, resembles that in the relation of geometry to real physical space. There was a time when the first essential of any geometry was that its properties should agree with those in real physical space. Physical space dominated geometry. Today, the relation is reversed; geometry exists in its own right, much richer than physical space in content, and able to represent any space found to have real existence. In the same way, system theory should, in my opinion, forget about physics and chemistry until it is stronger. (Ashby [1955] 1956)

Note, however, that the anti-substantialist accents of the English systemician were similar to Bertalanffy’s. Moreover, he was in line with the expositions of his colleague regarding the vocation of “systemology” as an autonomous and meta-scientific field, of which the constructions would be abstract and perfectly general. For that matter, one can also recognize in Ashby’s discourse (Ashby 1956, 1–2) the key element of Rashevsky’s philosophy of mathematizing, which had deeply influenced the Viennese systemician in the 1930s (Pouvreau 2013a, 536–544, 585–590).

Ashby’s set of problems focused on information processing in systems which are “closed” with regard to such processing. Nonetheless, he was clearly in line with Bertalanffy in his way of grasping organisms. He indeed only talked about “closeness” to information insofar as the cybernetic analysis was applied to the system formed by the organism *and* its environment. Both systemicians thus shared the general scheme according to which the former cannot be understood independently from its relationships to the latter – despite a different understanding of its meaning:

Given an organism, its environment is defined as *those variables whose changes affect the organism, and those variables which are changed by the organism’s behavior*. It is thus defined in a purely functional, not a material sense […] The free-living organism and its environment, taken together, may be represented with sufficient accuracy by a set of variables that forms a state-determined system. (Ashby 1952b, 36)

And it is to this system that Ashby applied the feedback concept:

The organism affects the environment and the environment affects the organism: such a system is said to have “feedback”. (Ashby 1952b, 36–37)

In doing so, however, Ashby chose a very wide definition of the feedback concept that in final analysis identified it with what may be called the “reciprocity of action”, where the specific meaning that “feedback” had by Wiener and his colleagues was lost.^30^ Interestingly, he was led to criticize this specific meaning based on holistic arguments partly fitting Bertalanffy’s:

When a circularity of action exists between the parts of a dynamic system, feedback may be said to be present […] To those whose aim is to get an understanding of the *principles* behind the multitudinous special mechanisms that exhibit them, “feedback” exists between two parts when each affect the other, as for instance, in *x*′ = 2*xy*, *y*′ = *x* – *y*² for *y*’s value affects how *x* will change and so does *x*’s value affect *y*. By contrast, feedback would not be said to be present in *x*′ = 2*x*, *y*′ = *x* – *y*², for *x*’s change does not now depend on *y*’s value; *x* dominates *y*, and the action is one way only. On the other side stand the practical experimenters and constructors, who want to use the word to refer, when some forward effect from *P* to *R* can be taken for granted, to the deliberate conduction of some effect back from *R* to *P* by some connection that is physically or materially evident. They object to the mathematician’s definition, pointing out that this would force them to say that feedback was present in the ordinary pendulum between its position and its momentum – a “feedback” that, from the practical point of view, is somewhat mystical. To this the mathematician retorts that if feedback is to be considered present only when there is an actual wire or nerve to represent it, then the theory becomes chaotic and riddled with irrelevancies. In fact, there need to be no dispute, for the exact definition of “feedback” is nowhere important. The fact is that the concept of “feedback”, so simple and natural in certain elementary cases, becomes artificial and of little use when the interconnections between the parts become more complex […] Such complex systems cannot be treated as an interlaced set of more or less independent feedback circuits, but only as a whole. For understanding the general principles of dynamic systems, therefore, the concept of feedback is inadequate in itself. What is important is that complex systems, richly cross-connected internally, have complex behavior, and that these behaviors can be goal-seeking in complex patterns. (Ashby 1956, 53–54)

The consequence of Ashby’s positions is that they blurred the overly radical distinction that Bertalanffy had made some years earlier between “dynamic order” and “machine order”. This is one first aspect of what we call Ashby’s “mediating” position and role between cybernetics and general systemology. Some other aspects of his reflections even more significantly met Bertalanffy’s, while making them more precise.

Ashby also referred to the importance of “isomorphism” (Ashby 1960, 85), e.g. between the modelling of the oscillating movement of a spring subject to a traction and to a friction force, and the modelling of the electric resonant circuit (including resistor, inductor and capacitor). In contrast to Bertalanffy, Ashby noted the existence of an “exact and objective definition” of isomorphism, in referring to the structuralist works of the French group of mathematicians named Bourbaki, with regard to what he described as “the most fundamental definition”.^31^ At the same time, he especially sought to formulate a definition “adapted to dynamic systems”. While interpreting the “canonical representation” of a “real machine” as the associated “transformation”, i.e. the specification of the set of “transitions” from one state to another one that represents it under the action of an “operator” (the set of factors acting on the state of the system), he gave the following definition:

The canonical representations of two machines are isomorphic if a one-one transformation of the states (input and output) of the one machine into those of the other can convert the one representation to the other. (Ashby 1956, 97–98)

Bertalanffy’s texts suffered from a lack of clarity in his use of the word “isomorphism” in reference to *partial* formal correspondences. In contrast, Ashby made a clarifying distinction between “isomorphic machines” and “homomorphic machines”, the latter ones expressing a “lesser degree of resemblance”:

Two machines may also be related by a “homomorphism”. This occurs when a many-to-one transformation, applied to the more complex, can reduce it to a form that is isomorphic with the simpler […] Two machines are homomorphic when they become alike if one is merely simplified, i.e. observed with less than full discrimination. Formally, if two machines are so related that a many-one transformation can be found that, applied to one of the machines, gives a machine that is isomorphic with the other, then the other (the simpler of the two) is a homomorphism of the first. (Ashby 1956, 97–103)

Ashby used this “weaker” concept to reformulate the problem of system modelling because systemicians generally only have incomplete information about the system they are investigating, particularly when dealing with biological systems. Considering that the terms “whole” and “partial” here refer to knowledge about the analytical determinations of the considered system and not to the system itself, Ashby was in fact in line with Bertalanffy’s perspectivist philosophy, but with more precise formulations:

The deliberate refusal to attempt all possible distinctions, and the deliberate restriction of the study of a dynamic system to some homomorphism of the whole, become justified, and in fact almost unavoidable, when the experimenter is confronted with the system of biological origin […] He must give up any ambition to know the *whole* system. His aim must be to achieve a partial knowledge that, though partial over the whole, is none the less complete within itself, and is sufficient for his ultimate practical purpose […] Science is not immediately concerned with discovering what the system “really” is, but with co-ordinating the various observer’s discoveries, each of which is only apportion, an aspect, of the whole truth […] The method of studying very large systems by studying only carefully selected aspects of them is simply what is always done in practice. Here we intend to follow the process more rigorously and consciously. (Ashby 1956, 106–107)

Ashby then could characterize a “model” as a “homomorphism” of a system defined on a “real machine”, and even more subtly as a system *M* of which a “homomorphism” *M’* is isomorphic to a “homomorphism” *S’* of the system *S* which has to be modelled; here, every aspect of *M* is not necessarily relevant for the representation of *S*, while all the aspects of *S* have not been taken into account for the modelling (Ashby 1956, 106–109).

Ashby wanted to model one particular form of adaptive behaviour: learning. Not every detail of his model would have a referent in the brain. The aim was to construct not only a formal model of the learning process, but also a “real machine” embodying this model. The behaviour of this machine would be a “homomorphism” of the learning process that manifests itself in real brains. Ashby called this machine the “homeostat”, referring to Cannon’s works. He actually constructed it in 1948.^32^ This construction was based on the interpretation of adaptive behaviour as the behaviour of a dynamical system all of whose “essential variables”^33^ are maintained “within their normal limits” (which define in its space phase a “stability area” of the system) (Ashby 1952b, 58–64). According to Ashby’s behaviourist point of view, this behaviour was simultaneously interpretable as the result of a “trial and error” process, the “error” corresponding to the leaving of the “stability area”.^34^ Understanding adaptation thus became understanding how the system can reconcile both the requirement for stability of its “essential variables” and the requirement for adequate change according to environmental stimuli. Ashby’s response involved the concepts of “step-functions” and “step-mechanisms”: a dynamic system can satisfy both those requirements while being “state-determined” if some of its variables, the “main” ones, behave as “step-functions”. Thus, the whole set of those variables has not only one, but several “fields”. Moreover, if their respective changes overcome definite step-values, the system is forced to leave its “stability area” in one given “field” of the main variables and enter a “critical state” (Ashby’s “step-mechanism”) (Ashby 1952b, 87–94). The clear originality was that Ashby sought to overcome the dispute between determinism and spontaneism in giving a meaning to the concept of a “machine that can be at once determinate and capable of spontaneous change”.^35^ Such a machine had to be what he called an “ultrastable system”, designed on the basis of a double feedback mechanism (Ashby 1952b, 98, 108). With the homeostat, Ashby moreover exposed a cybernetic mechanism explaining this amplification ability that had been constitutive of Bertalanffy’s and Mittasch’s reflections on “impulse causality” [*Anstoβkausalität*] in the mid 1930s (Mittasch 1938; von Bertalanffy 1937, 34, 1949b, 161–164).

The most important issue, however, was probably less in these various results and hypotheses than in a general law to the formulation of which Ashby was led while furthering his reflections upon the “homeostat”, after its construction. This machine clearly demonstrated that a system with a sufficiently rich repertoire of alternative configurations can remain stable despite strong and highly variable environment perturbations: the precondition is its ability to reconfigure itself in at least as many different ways as required by these perturbations. Ashby recognized an underlying very general principle, which he formulated and published in 1956.

Ashby defined the “variety” of a set as the base 2 logarithm of the number of its different elements; in particular, the variety of a dynamic system was defined as the base 2 logarithm of its different states (Ashby 1956, 124–126). This quantity was thus related to information theory. An associated concept was “constraint”, which refers to the possibility to map one set on another one with a lesser “variety”.^36^ Ashby’s “law of requisite variety” is a general law of regulation which constitutes a mathematical theorem in the framework of a formal theory of control. This law states that a system *R* can control (or regulate) another system *S*, that is, lessen its “variety” while maintaining its stability, if, and only if *R*’s variety is higher than *S*’s variety: “only variety can destroy variety”. Another form of this law, which is related to one of Shannon’s theorems,^37^ is that “*R*’s capacity as a regulator cannot exceed its capacity as a channel of communication” (Ashby 1956, 206–211, Ashby 1962, 273–277). This implies that the “variety” of *S*’s possible responses, therefore also of its structure and functions, must be all the higher to enable its regulation because this system is subject to a high “variety” of perturbations and because it has few “acceptable” states (i.e. maintaining its essential variables within the limits that are required in order to ensure its “survival”).^38^ This law superimposes a fundamental limit to the possibility of regulation. It also implies that the more information is provided by *R* to *S*, the less is *R*’s control ability (that is, also its efficiency). This is valid until a situation in which *S*’s “variety” becomes higher than *R*’s, *S* thus becoming likely to control *R* (a cybernetic reinterpretation of the Hegelian dialectics of the master and the slave, which predicted in abstract terms the possibility of a control of humans by their machines). Ashby pointed out the importance of his law in biology:

A species continues to exist primarily because its members can block the flow of variety (thought of as disturbance) to the gene-pattern, and this blockage is the species’ most fundamental need […] By the law of Requisite Variety, the amount of disturbance that reaches the gene-pattern can be diminished only by the amount of information so transmitted. That is the importance of the law in biology. (Ashby 1956, 212)

More generally, the scope of this law would concern all scientists working on the issue of control, especially those dealing with systems having a high “variety”.

Two major convergences between Ashby and Bertalanffy are evident and further support the mediating position of the former between the latter and cybernetics. The first is that the “law of requisite variety” was a perfect example of an “exact general system law” whose possibility Bertalanffy had claimed and whose investigation he had promoted. This “law” was quickly recognized as a paradigm among systemicians. More than twenty years after being stated, it was even raised (with some exaggeration) to the rank of “the” *only* statement deserving the true status of general system “law” (in the deductive-nomologic sense of the term).^39^ The second convergence deals with the necessity to grasp the organism and its environment as a system, accounting for their interactions in their reciprocity. Some Ashbian formulations certainly give the impression of an opposition to Bertalanffy’s principle of “anamorphosis” – a trend towards a rising complexity as found in evolution (cf. Pouvreau and Drack 2007, 310). An example is his principle of “decay of variety”. Ashby pointed out its congruence with the second principle of thermodynamics in order to demonstrate the generality of his own principle: “as time progresses the variety in the set cannot increase and will usually diminish” (Ashby 1956, 136). Bertalanffy, however, was well disposed towards such a statement because the set to which it must be applied is closed from every point of view. Ashby was explicit on that point in 1962: his whole cybernetics justified the idea that a “self-organization” is impossible in a system which does not interact with its environment, the organizational changes necessarily originating outside the system. He also criticized the relevance of the “self-organization” concept itself, which is according to him “self-contradictory” *even if it is used precisely* ^40^: the so-called “self-organized” system always has to detect – by means of its exchanges with its environment, in the form of perturbations affecting its “essential variables” – the “variety” of this environment. It can, by itself, gain some “variety” only if it collects information about this environment or if it attempts to control the matter and energy exchanges it has with the latter. The theory of “variety” finally brought a logico-mathematical foundation to the idea that originated from Fechner and which Bertalanffy opposed to Schrödinger as early as 1949. Accordingly, the “organismic” order should not be thought of as “order from order”, but as “epigenetically” emerging out of chaos based on principles inherent in dynamic systems. Ashby gave a significant impulse to what was to become, notably with Prigogine and Atlan, the famous principle of “order from noise” (Atlan 1979, Prigogine and Stengers 1979). Note here that Ashby proffered as early as 1952 (having in view the organism as well as the “homeostat”) that “chaotic information is by no means useless, but is, in fact, perfectly useable *provided that the machine has been designed to make the necessary selection*” (Ashby 1952a, 53).

Notwithstanding all those significant lines of convergence, Ashby (1958, 2) distinguished two main lines towards “general system theory” (more precisely, towards “basic theoretical systemology”). The first was the method of Bertalanffy and co-workers, which he held to be essentially empirical. The second, to which Ashby himself was devoted, considered “the set of ‘all conceivable systems’ and then reduc[ed] the set to a more reasonable size” (see also Ashby’s criticism on Bertalanffy’s method mentioned above). In other words, Ashby considered himself as working deductively, Bertalanffy inductively. von Bertalanffy (1962, 4) acknowledged this analysis of the two ways,^41^ both having advantages and shortcomings. He also admitted that he intuitively came up with the main principles of wholeness, sum, centralization, differentiation, leading part, closed and open system, finality, equifinality, growth in time, relative growth and competition. Nonetheless, Bertalanffy did not oppose a deductive approach: even in his first works on theoretical biology, he rather emphasized it (cf. Pouvreau and Drack 2007). With regard to Ashby, he noted that “there is no royal road to General Systems Theory. As every other scientific field, it will have to develop by an interplay of empirical, intuitive and deductive procedures.” (von Bertalanffy 1962, 6; see also Pouvreau 2014a, 202–205). In fact, Bertalanffy’s approach can also be said to be deductive, even if it is not as abstract as Ashby’s. Within GST, certain system laws are derived from the definition of a system as a “complex of components in mutual interaction” (von Bertalanffy 1965b, 1100). This is thus a case where, although alleged, no clear differences can be detected between GST and cybernetics.

If cybernetics, as understood by Ashby, started with the most general systems in order to deduce all possible instances, then cybernetics would be the larger field and include GST. Bertalanffy, however, criticized Ashby’s approach because even though it was deductive, it left out an important issue: systems that show progressive differentiation and evolve in that way to states of higher complexity. This can be found in biological ontogenesis and phylogenesis, as well as in social organizations (von Bertalanffy 1962).

### 4.5. Cybernetic concepts and methods in line with general systemology

Both approaches – cybernetics and general systemology – aim at generalized models, i.e. at formal constructions that can be used to investigate entities relating different components by means of their representation as “systems” (cf. von Bertalanffy 1970, 24); or even to construct “systems” of entities whose components are unknown. Thus isomorphic problems, i.e. problems whose structure of concepts can be accounted for by a single formalism, can be tackled and transdisciplinary models can be established (Pouvreau 2014a, 216–217).

In 1964, Young surveyed the frequency of concepts used by various authors concerned with “systems” (Young 1964). Among the 39 authors from 11 disciplines considered, he outlined 36 frequently used concepts. The cluster analysis shows that Bertalanffy shares 18 concepts (out of the 22 concepts he employs) with Ashby and 12 with Wiener, among them homeostasis, adaptation, feedback, and open and closed systems. According to Young’s study, the following concepts have been used by Ashby, but not by Bertalanffy: boundaries and environment, learning, change, isolation and interaction, overload, repair and reproduction, field, and state-determined system. Similarly, the following concepts would allegedly have been used by Wiener, but not by Bertalanffy: boundaries and environment, communications, integration and differentiation, learning, goal, isolation and interaction, overload, repair and reproduction, and decay. Although such a cluster analysis is interesting, the details in this case are misleading. Concepts such as boundaries and environment, integration and differentiation, goal, isolation and interaction, repair and reproduction, field, and decay were definitely used – perhaps with different emphasis compared to Ashby and Wiener – in von Bertalanffy (1969a) and other works in his earlier organismic biology (Pouvreau 2013a). This points to an even greater overlap of the concepts used by Bertalanffy and the cyberneticists.

Ashby is rightly seen as “[p]erhaps the most important person” in cross-fertilization between general systems research and cybernetics (Klir 2001, 49). A survey among system researchers, published in the late 1970s (Klir 1978, Appendix B), revealed that “Ashby was by far the most influential person in [the] systems movement”. According to that survey, Ashby had influenced about twice as many researchers as Bertalanffy (the second most influential person), and about three times as many as Wiener and Rapoport – the third most influential persons (Klir 2001, 49).

### 4.6. Do open systems and feedback systems involve different types of regulation?

Bertalanffy constantly emphasized the distinction between regulation in dynamic systems and in feedback systems. The original negative feedback mechanism – not the extended version of Ashby discussed above – is used to minimize the value of a variable (e.g. the distance of one object to another). The arrangement that performs such an operation is fixed and pre-established. If the system is disturbed, the feedback mechanism drives the value to a minimum again. Regulation against disturbance in a dynamical system is different from such a fixed or frozen (*erstarrt*) feedback system. But dynamic order (which Bertalanffy held to be the primary form) can freeze into a (secondary) order with increasingly segregated elements without interaction and fixed feedback loops. A system tending towards such a secondary order appeared as increasingly mechanistic to Bertalanffy. As far as an order is “organismic” in his sense, the dynamics is however always primary, whatever its level of mechanization. For example, when one biological process is altered in an organism, this dynamical system develops compensation strategies. Bertalanffy held that “primary regulations” are a result of dynamic interactions and can be superimposed by secondary regulation where the structures are fixed and “machine-like”. Accordingly, a dynamical order exists before any structural order in the organism. This enables a development towards mechanization, potentially leading to one-way causality, physically speaking to a reduction in the degrees of freedom. And this may be applied ontogenetically as well as phylogenetically (von Bertalanffy 1951a, 360):

Dynamic regulation precedes structural feedback. Consequently, “cybernetic” models particularly apply to “secondary” regulation, but “kinetic” models are required for “primary” regulations. Thus (speaking broadly), regulation of metabolism in the cell (e.g. in respiration or photosynthesis) is based upon a network of reactions in an open system; with progressive evolution, ever more elaborate homeostatic mechanisms, such as nervous and hormonal feedbacks, arise. (von Bertalanffy 1967, 67–68)

Concepts and models of equilibrium, homeostasis, adjustment, etc., are suitable for the maintenance of systems, but inadequate for phenomena of change, differentiation, evolution, negentropy, production of improbable states, creativity, building-up of tensions, self-realization, emergence, etc.; as indeed Cannon realized when he acknowledged, beside homeostasis, a “heterostasis” including phenomena of the latter nature. (von Bertalanffy 1969a, 23)

In general the homeostasis scheme is not applicable (1) to dynamic regulations, that is, regulations not based upon fixed mechanisms but taking place within a system functioning as a whole (for example, regulative processes after brain lesions), (2) to spontaneous activities, (3) to processes whose goal is not reduction but building up of tensions, and (4) to processes of growth, development, creation, and the like.^42^ (von Bertalanffy 1965b, 1102–1103)

Bertalanffy also stressed that dynamic principles are necessary to study psychological phenomena, e.g. while the central nervous system is developing towards an increasingly mechanized state, and to study behavioural sciences, e.g. as plastic and conscious activities become fixed and unconscious (von Bertalanffy 1951b, 26ff). According to him, machine models can especially be applied for the segregated and mechanized state, but hardly for the developing stage. Moreover, inventiveness or creativity cannot be tackled with the concept of a brain as a calculating machine. Bertalanffy stated that

[…] it is perhaps the profoundest objection against Cybernetics […] that “thinking” proper, and the corresponding neural mechanism, is not a primeval function, but rather a late product of evolution. (von Bertalanffy 1951b, 31)

As Ungerer (1966, 125–126) reported, the psychologist Wolfgang Köhler (1955), one of the leading gestalt theoreticians, also pointed out the contrasting regulation between an open system in flux equilibrium [*Fließgleichgewicht*] in an organism and the fixed arrangement in the material structure of an automaton. The anatomical structures where the regulatory processes take place are – in contrast to the structures of an automaton – themselves continuously changing through metabolism. Not only the “information” flow in the regulation is dynamic, but also the material structure that conveys and processes the information is dynamic (open system).

In certain cases, the results of the different types of regulation are similar. Bertalanffy, however, insisted on the difference between these types, even though no sharp border can be drawn:

Even though the result of regulations based on a flux equilibrium in an open system and those which are based on homeostasis [in Wiener’s sense] are similar, and even though both types of regulation overlap in many physiological phenomena, one should clearly distinguish them. The maintenance of flux equilibrium in an open system is based on kinetic and dynamic principles. Whereas homeostasis is based on mechanisms that are typified by feedback; i.e. part of the output of a machine is – as information – guided back to the input and regulates the input and stabilizes the performance of the machine. (von Bertalanffy 1953a, 38–39)

It seems that the *primary* regulations in a living organism are the result of a dynamic interaction within a unified system according to general system laws. Superimposed on them are *secondary* regulations, which are dominated by “machine-like” mechanisms, in particular those of feedback character. There is of course no strict border between “dynamic systems” and “machines”. The essence of a machine consists in restricting conditions imposed upon a system of force, which lessen the number of degrees of freedom, in general such as only one kind of movement possibility remains. But even in the man-made machines there are many kinds of gradation between rigid and flexible coupling, which only allow one or several definite degrees of freedom. In the field of biology, primary regulations appear as the ones of a type of system which is less constrained, so that a higher degree of dynamic interaction and freedom remains. According to the “principle of progressive mechanization” constraints develop – ontogenetically and phylogenetically – which on the one hand increase the performance of the system, but on the other hand, limit the capacity of primary regulatory, the latter makes a recovery possible even after severe damage or loss of entire parts of the material system. (von Bertalanffy 1953a, 38–39; cf. also von Bertalanffy 1962, 7–8)

The reference to the “machine-theory” of cybernetics concerned its type of explanation (which is related to a type of causality). These explanations persistently relied on “pre-established structural arrangements”, to which Bertalanffy opposed the principles of his “open system in flux equilibrium”:

In both cases [cybernetic and open systems], the system develops forces directed against the disturbance coming from outside and tending to re-establish a normal state. But the causal basis is different. According to the system conception, “teleological” behavior results from dynamic interaction within a unitary system that attends certain conditions of equilibrium or steady state. The compensation of disturbances coming from outside is the result of kinetic and dynamic counteraction of the type of Le Chatelier’s principle, which can be expanded and generalized also to open systems. According to the feedback scheme, “teleological” behavior, i.e. a behavior either maintaining the system itself (homeostasis) or attaining a goal outside the system is due to pre-established structural arrangements. The model scheme in feedback is essentially a machine theory. (von Bertalanffy 1951a, 353)

Bertalanffy admitted that (first order) cybernetics provides a “refined machine theory”, but the essential element for him was that it could “hardly be conciliated with definite facts indicating an authentic dynamic order” (von Bertalanffy 1951b, 27). His judgment was based on another argument taking the form of a radical criticism. It consisted of challenging the idea according to which the feedback schemes – in consequence of their inherent circularity of information transmission and because of the possibility to combine them – can account for the holistic causalities characterizing complex systems:

Feedback mechanisms are essentially one-way causal chains. This is not altered even in the case of what may be called compound feedback, i.e. when not only one, but some regulating mechanisms are active, as in the case, for instance, in ship-steering systems. Feedbacks are what has been called by H. Jordan “amboceptors”. In many biological regulations, however, we have pantoceptor reactions, i.e. interactions of many or all elements integrated in the system. That dynamical order is not taken into consideration by cybernetics, is made clear in an earlier publication (Rosenblueth, Wiener, and Bigelow [1943]). (von Bertalanffy 1951a, 354)

Speaking in epistemological terms: while the prototype of undirected physical processes is *linear causality* (cause A being followed by effect B), the cybernetic model introduces *circular causality* by way of the feedback loop and this makes for the self-regulation, goal-directedness, homeostasis, etc., of the system. In contrast, the more general system model is that of *dynamic interaction* between many variables. (von Bertalanffy 1967, 67; italics in the original)

Bertalanffy was justified in stating that the dynamical order of “open systems in flux equilibrium” was not really taken into account by the cyberneticists (although some of them, notably Cannon and Wiener, had understood and stressed the fundamental importance of openness from the thermodynamic point of view). He was, however, mistaken to state the reproach that cybernetics is “restricted to closed systems”. The root of this mistake is the evocation by McCulloch of the possibility to explain the *telos* of “cybernetic machines” “in terms of activity in closed circuits”: at that time, Bertalanffy confused closeness to information with closeness to matter and energy exchanges (von Bertalanffy 1951a), although such machines are open to such exchanges (cf. Richardson [1991, 122, 298], who points to the distinction between materially closed and causally closed systems).

Typical feedback or homeostatic phenomena are “open” with respect to incoming information, but “closed” with respect to matter and energy. (von Bertalanffy 1969a, 163)

[Ashby’s] “modern definition” of system as a “machine with input” supplants the general system model by another rather special one: the cybernetic model, i.e. a system open to information but closed with respect to entropy transfer. (von Bertalanffy 1962, 5)

Cybernetic systems are “closed” with respect to exchange of matter with environment, and open only to information. For this reason, the cybernetic model does not provide for an essential characteristic of living systems, whose components are continually destroyed in catabolic and replaced in anabolic processes, with corollaries such as growth, development and differentiation. (von Bertalanffy 1967, 68)

Bertalanffy recognized two basic distinctions between cybernetics and GST in the “narrow sense” (i.e. “organismic systemology”), namely the machine concept versus the organismic concept and the closed versus the open system concept:

Here the two basic features of the theory are obvious, namely, first the machine model of biological phenomena, secondly, the restriction to closed systems. In this sense, our theory seems to be more revolutionary, since, first, it overthrows, as far as basic phenomena are concerned, the Cartesian machine-theory of the organism, and, secondly, is based upon the conception of the organism as an open system. (von Bertalanffy 1951a, 354)

Of course, feedback-steered machines also allow for material and energy flow like in open systems but, according to Bertalanffy, other implications of the open system concept such as growth and development cannot be tackled with feedback based on a given structure alone.

For the same reason [system closed with respect to exchange of matter], a cybernetic system cannot be “self-organizing”, that is, evolving from a less to a more differentiated state. To be sure, cybernetic systems provided with memory devices can learn, that is, change and increase their organization owing to information input. They cannot undergo processes of differentiation which require input of energy (and matter). In other terms, cybernetic systems can only increase in their entropy content and decrease in information content, information being partly converted into noise but not vice versa (Shannon’s Tenth Theorem). Anti-entropic processes presuppose the system’s being “open”, that is, transfer of matter and energy taking place. (von Bertalanffy 1967, 68)

The openness to information is exactly the opposite of Ashby’s understanding (see the section on first order cybernetics). The distinction between open and closed depends where the borders are drawn. For Ashby, the system plus the environment is information tight. He insisted on the fact that the closed system is “organism plus environment” (Ashby 1962; cf. also Ashby 1952b, 16, 40). Moreover, Rosen (who developed his own formalism^43^) legitimately criticized Bertalanffy’s allegations:

More serious, in the reviewer’s opinion, and in fact partly comprising the only important shortcoming in the book [von Bertalanffy 1969a], are the repeated distinctions made between dynamical systems and systems with feedback, and between dynamical system theory and automata theory. For it is one of the major insights of dynamical system theory that the distinction between feedback control systems and dynamical systems is only one of emphasis, both kinds of system being essentially comprised within the mathematics of stability theory; likewise it is another important insight drawn from system theory that automata theory, far from being different in kind from dynamical system theory, is actually as close a paraphrase to a discrete setting of the formalism of dynamical system theory as possible. These are very important unifying insights, and come from the technical development of system theory itself. (Rosen 1969)

Details on stability and how feedback systems and dynamical systems can be treated with a single formal set-theoretical framework can be found in Mesarovic and Takahara (1975, chapters IX, X)^44^. Furthermore, a classification of systems and the connection of systems to finite automate are treated in Mesarovic and Takahara (1989, 5–6, 16, 45–46, 119–121, 139–140, 142). Bertalanffy seems to have acknowledged Rosen’s criticism, which is apparent in a revised edition of the book that Rosen reviewed. In a new appendix, Bertalanffy stressed that the formalisms do not oppose each other. If Ashby’s broad definition of feedback had been taken into account, this issue could have been resolved earlier.

As mentioned, internal and external descriptions largely coincide with descriptions by continuous or discrete functions. These are two “languages” adapted to their respective purposes. Empirically, there is – as emphasized in the text – an obvious contrast between regulations owing to the free interplay of forces within a dynamical system, and regulations owing to imposed constraints by structural feedback mechanisms. Formally, however, the two “languages” are related and in certain cases demonstrably translatable. For example, an input-output function can (under certain conditions) be developed as a linear *n*th-order differential equation, and the terms of the latter considered as – formal – “state variables”; while their physical meaning remains indefinite, formal “translation” from one language into the other is thus possible. (von Bertalanffy 1969a, 255, also: 1972a, 34)^45^

Similarly, predator-prey systems, usually described dynamically by Volterra equations, can also be expressed in terms by cybernetic feedback circuits. (von Bertalanffy 1969a, 256)

Different models might be used for a single process, potentially emphasizing different aspects of the latter. Conversely, one single type of model may be applied for qualitatively different processes. In such a case however, this does not change the nature of the considered processes.

The two ways of describing, dynamic systems theory and cybernetics, fall largely together with *internal or external description*. In the first case one tries to describe the system behavior by dynamic equations of motion, which are typically simultaneous differential equations between system variables. In the second case, the external description, the system is considered as a “black box”, from which only the […] relationship between input and output is known; in general they are described by discontinuous transfer functions which, in self-regulating systems, contain feedback loops. As mentioned, dynamic regulation, for example by equifinality in an open system, and self-regulation by *feedback* mechanisms are different in their model, in the empirical procedure, in the mathematical description. At least in certain cases it can be shown that these descriptions are equivalent or isomorphic, where, so to speak, one can be translated into the other. (von Bertalanffy 1972b, 26)

To sum up: what Bertalanffy definitely confused is the openness or closeness of a cybernetic system. The other issues are not contradictions or confusions, but rather an evolution and precision of his basic ideas. The fact that both types of models can be accounted for in the framework of a unified mathematical theory of stability (as noted by Rosen and acknowledged by Bertalanffy) does not change the fact that these models are epistemologically different. They differ with respect to the relevant and important dichotomies internal/external and continuous/discrete, as well as with respect to the formalisms, except perhaps in Ashby’s view of feedback. Moreover, they may be relevant for different aspects of a single phenomenon under study. In line with general systemology (and its perspectivism), these different aspects and their models may have a common underlying basis, whose layout can be revealed by a single general model. Different applications, however, can emphasize different aspects. This does not mean that the aspects and their models are identical and should be confused. Thus, Bertalanffy’s dichotomies remain relevant, although Rosen was right in moderating them.

### 4.7. Teleology

Coming from philosophy and biology, Bertalanffy crossed the problem of teleology very early (Pouvreau and Drack 2007). Nevertheless, the core scheme of teleology in cybernetics (see the subsection on first order cybernetics) was also important for him, and he considered the question “in how far the [cybernetic] model proposed may serve as an explanation of biological teleology” (von Bertalanffy 1951a, 350). In both the concept of cybernetics and the theory of open systems, “forces directed against the disturbance coming from outside” (von Bertalanffy 1951a, 353) are employed. Nonetheless, their causal bases are different (see previous subsection).

The first step towards a detachment of his understanding of teleology from that of the cyberneticists was made in 1950. It involved the classification of “types of finality” (von Bertalanffy 1950, 159ff, reprinted in 1969a, 77ff). This took into account cybernetics and thus refined a tripartition that he had constructed the year before (von Bertalanffy 1949a, 125). At that time, he had distinguished only cases (2)(i), (2)(iii) and (2)(iv) of Table 1.

**Table 1.** Bertalanffy’s classification of teleology; after von Bertalanffy (1950, 159ff; reprinted in 1969a, 77ff).

1 – *Static teleology* (fitness)	2 – *Dynamic teleology* (*directiveness of processes*)			
	(i)	(ii)	(iii)	(iv)
	Direction of events towards a final state expressible as if the present behaviour were dependent on that final state	Directiveness based upon an arrangement of structures	Equifinality (independence of the final state from the initial conditions and from the ways taken to reach it)	True finality (purposiveness); determination of the actual behaviour by the foresight of the goal

von Bertalanffy (1950, 159ff, reprinted in 1969a, 77ff) distinguished two major types of finality, namely static and dynamic teleology (see Table 1). “Static teleology” refers to “an arrangement that seems to be useful for a certain ‘purpose’”, such as fur for keeping the body warm: Bertalanffy, who apparently restricted this type of teleology to the organic realm, considered that it can be explained by the synthetic theory of evolution (random mutations and natural selection). His “partition” of the cases of “dynamic teleology” – “meaning directiveness of processes” – into four subclasses was not perfectly clear. This is because it did not represent a logical disjunction. A case in point is type (2)(iii): in definite cases such as his own theory of global animal growth, it can be viewed as a particular case of (2)(i), where he reunited all cases where the system reaches a final state independent of time. This approach enabled him to dismiss once again vitalism as an attempt to explain teleology types (2)(ii) and (2)(iii) by type (2)(iv). A main point here is that this classification enabled him to introduce, with types (2)(ii) and (2)(iii), a dichotomy between the cybernetic model of systemic regulation and his “organismic” model of regulation based upon the “open system” concept. On the one hand, Bertalanffy identified the type (2)(iii) “dynamic teleology” to be the one manifested in open systems, whose development is oriented toward a “flux equilibrium”. He did this by stressing the fact that such a teleology is based upon the “dynamic interaction of the elements of the system” and not upon “predetermined structures or mechanisms” (von Bertalanffy 1950, 160, 1951a, 352–353). On the other hand, he classified all feedback mechanisms in type (2)(ii).

Note that first order cybernetics was mostly concerned with subclass (2)(ii), although a difference was seen between the man-made machine and the organism. The machine works in “a way to yield certain products and performances”, whereas in the organism the order of processes “is such as to maintain the system itself”. In the latter, homeostasis comes into play as “an important part of these processes”. Type (2)(iii), equifinality, i.e. the final state can be “reached from different initial conditions and in different ways”, is found in open systems and is responsible for “primary regulability”. Static teleology and dynamic teleology of type (2)(ii) are found in man-made machines and are due to the “planning intelligence”, which in turn is of type (2)(iv) (von Bertalanffy 1950, 159ff, reprinted in 1969a, 77ff).

Bertalanffy’s critical argument, repeated several times between 1950 and 1953, involved stressing the fact that the cybernetic schemes of regulation by feedback fail to account for the regulation processes corresponding to his dynamic teleology type (2)(iii). In a first step, he strived to demonstrate the impossibility to subsume the whole set of organic regulation phenomena under the “homeostasis” concept. He did this by denouncing the trend to conceptualize in that way processes which either have nothing to do with feedback mechanisms (such as metabolic regulation and organic growth) or cannot be completely reduced to such mechanisms (such as the regulations of oxygenation and blood acidity). According to Bertalanffy, the frequent “loose” use of the term “homeostasis” implies a loss of its “proper meaning”, as well as significant epistemological dangers bound to inappropriate methodological procedure:

Where we have homeostasis, we must unveil the mechanisms involved. But where we have purely dynamical regulations, the consideration of phenomena as “homeostasis” leads us astray since we look for mechanisms where there are none. (von Bertalanffy 1951a, 357)

Bertalanffy pointed more widely to the phenomena which had fostered, in the 1920s and 1930s, the rise of holistic physiologies, medicines and psychologies. These phenomena considerably influenced his early reflections (Pouvreau 2013a, 136–208):

This applies to the classical “proofs of vitalism” as advanced by Driesch and others, such as equifinal regulation in embryonic development, self-regulation in metabolism, “remaining a whole” in spite of division in biological elementary units such as genes and chromosomes, as well as to regeneration, wound-healing, regulations within the nervous system after injuries in its peripheral and central parts, re-establishment of gestalt-perception, etc. Feedback gives no more an explanation of these phenomena than any other machine-theory, whilst these features are, at least in their general aspects, consequences of the open-system character of the organism. (von Bertalanffy 1951a, 355–356)

Goldsmith (1981) rightly points out that the distinctions Bertalanffy made would have helped considerably to overcome misunderstandings towards teleology, also concerning cybernetics. Furthermore, she states that squabbles such as those between the philosopher Richard Taylor and the cyberneticists Rosenblueth and Wiener are unnecessary. Taylor (1950) contested that the concepts of purpose and teleological behaviour such as defined by Rosenblueth et al. (1943) could serve the claimed objectives or be useful: the criterion for deciding whether a behaviour is directed towards a “final condition” or not would be arbitrary and their characterization of “purposeful” and “teleological” behaviours would be so wide that they would encompass all types of behaviour. Rosenblueth and Wiener (1945) in parallel identified scientific knowledge with “a sequence of abstract models”. These authors aimed at “replacing the considered part of the universe by a model of similar but simpler structure” and acknowledged the perspectivist character of such constructions. Rosenblueth and Wiener (1950) rejected the criticism by Taylor and were opposed to his statements that “[t]he analysis of any process can be carried out in many different ways that are scientifically valid and useful”; that the notion of purpose is, among others, a legitimate “fundamental category”; and that although its validity is limited, such a limit cannot be an argument for denying the virtues of teleological approaches. In the latter case, this is because “the adoption of a teleological approach simplifies the analysis of goal-directed behavior and enlarges the scope of this analysis”, without implying any philosophical belief to final causes. A further critique from philosophy came from Hans Jonas: in cybernetics, “having a purpose” (*einen Zweck haben*) was mistaken with “serving a purpose” or “carrying out a purpose” (*einen Zweck ausführen*), and the first meaning was reduced to the second one; this is a mistake that Bertalanffy did not make (Tibaldeo 2008).

From Bertalanffy’s point of view, there was also no contradiction between determination and teleology, which is clear in his early statement: “What in the whole denotes a causal equilibrium process, appears for the part as a teleological event” (von Bertalanffy 1929a, 102, 1929b, 390). Teleology (or holistic approach) and causality (or meristic approach) were regarded by Bertalanffy as complementary (in a Bohrian sense) modes of a formal description, which can both claim scientific legitimacy as far as they are de-anthropomorphized. From the viewpoint of the history of thinking about teleology, there was a change with Kant’s *Kritik der Urteilkraft* [Critique of Judgement], which was transmitted to Bertalanffy mostly by Ungerer’s habilitation thesis (Pouvreau 2013a, 182–188, 193–194, 455–463). Rosenblueth et al. (1943, 24) argued similarly in stating that teleology does not oppose determinism.

## 5. Beyond science – towards ideology and world view

Without distinguishing the two strands resulting from GST and cybernetics, Gray (1981) pointed to the humanistic concerns in the overall field and discussed how they still influence thinking. Wiener and Bertalanffy had already strongly emphasized the ethical issues their models touch (e.g. Wiener 1954; von Bertalanffy 1967). We therefore discuss this important issue below.

### 5.1. Mechanicism and reductionist biases

The bias that cyberneticists usually shared was less a mechanicism (in the sense of a meristic approach to phenomena associated with determinism, reductionism and/or reactivism)^46^ than a reductionism transforming the old “iatromechnicism” based on a sophisticated “machine” concept. Bertalanffy was in that regard correct in noting that the interpretation of brain functioning based on the model of the “computation machine” was a “continuation of the development initiated by the iatromechanicists” (von Bertalanffy 1965a, 292):

We believe that *men and other animals are like machines from the scientific standpoint because we believe that the only fruitful methods for the study of human and animal behavior are the methods applicable to the behavior of mechanical objects as well*. Thus, our main reason for selecting the terms in question was to emphasize that, as objects of scientific enquiry, humans do not differ from machines. (Rosenblueth and Wiener 1950, 326; our italics)

The Macy conferences as well as contemporary papers on cybernetics bathed freely in this reductionism, including representatives of social sciences such as Gregory Bateson and Karl W. Deutsch: life was understood as an entropy reduction device; the organisms and their associations, particularly humans and their societies, as servomechanisms; and the brain, as a computer (Heims 1991, 27–28).

Criticisms of this reductionism appeared almost at once, as early as the first half of the 1950s. The English philosopher John O. Wisdom was among the first, in line with Ralph W. Gerard (whose criticism addressed the fact that the formal model of the neuron network neglected the role of chemical concentrations and continuous electric fields: see Heims [1991, 21]). Wisdom pointed to the fact that, in opposition to Warren McCulloch’s and John von Neumann’s pretensions, cybernetics could not be viewed as the basis of neurology, but only as one of its branches. This was because many neurophysiological processes do not involve any feedback mechanism.^47^ Cyberneticists sought, for example, to widen the application spectrum of their concepts and principles to behavioural sciences. Wisdom criticized the particularly excessive character of such a widening:

The Newtonian machine was not an adequate model of a human being. The question now arises whether the cybernetic model is adequate. Clearly it is not; there are too many ways in which any machine that can be envisaged at present fails to answer to human behavior […] It will doubtless seem to many that cybernetics explains purposive behavior in such a way as to leave the mental aspects of action out of account, that it thereby gives the *coup de grace* to the mind, and bears out the behaviorist and physiological approach. Now it is true that cybernetics leaves out the mental aspects of action. But the proper inference from this is that cybernetics is in the tradition of physiology and neurology. It does not solve the problem of the relation of mind and body, because it omits one of the parties concerned and suffers from the one-sidedness of its ancestors. (Wisdom 1951, 22–23)

The most violent criticism at that time came from the French philosopher Raymond Ruyer. Without denying the practical and theoretical interest of cybernetics, he attacked the “fallacious character of its mechanistic postulates”. This would be expressed by the “purely apparent and superficial character of the success of its mechanical models” and would be deep-rooted in a “failure to understand the origin of information” (Ruyer 1954, 235–236). It would be based on a paradox which ultimately consists of admitting a kind of “perpetual movement of the third kind”, a paradox which reduces cybernetics to absurdum. Combining the principle according to which cybernetic machines cannot win information and the hypothesis according to which brains are cybernetic machines of the same kind as artefacts would make impossible any information creation: a truly cybernetic world would be a world of “pure transmitters”. Even if Ashby’s work on “self-adaptive” systems (Ashby 1952a) weakened its relevance, this criticism was typically symptomatic for giving rise to serious and wide-ranging doubts on the applicability of cybernetics outside the field of engineering, particularly to biology:

It would be as much dangerous to believe blindly in the models provided by cybernetics as to disdain them […] Psychologically, the integral faith of the cyberneticists in their models looks like the faith of the researchers in the perpetual movement or to the quadrators of the circle. It may be temporarily useful because of the enthusiasm that it raises, but it can quickly become harmful […] All the internal difficulties of cybernetics originate in the same mistake in principle and in the unfortunate postulate according to which the information machines are always supervised by a conscious and meaningful activity […] Every genuine anti-chance is the basis of connections and every connection is based on consciousness. Applying to the machine in order to clear up the mystery of anti-chance and the origin of information is therefore a contradiction. Organic and psychological information, the ordered and meaningful epigenesis of structures in memory and invention cannot be explained mechanistically. (Ruyer 1954, 21, 24, 81, 141, 235)

Bertalanffy also criticized the so-called “mechanistic” approach of cybernetics, pointing to the ethical dimension of his own program and stressing the potential danger that the system approach bears:

This humanistic concern of general system theory as I understand it makes a difference to mechanistically oriented system theorists speaking solely in terms of mathematics, feedback and technology and so giving rise to the fear that system theory is indeed the ultimate step towards mechanization and devaluation of man and towards technocratic society. While understanding and emphasizing the aspect of mathematics, pure and applied science, I do not see that these humanistic aspects can be evaded if general system theory is not limited to a restricted and fractional vision. (von Bertalanffy 1969a, xxiii)

But he also acknowledged that cybernetics is not “mechanistic” in the sense that it is not meristic: the behaviour of entities of the phenomenal world cannot be described comprehensively with the sole knowledge of the properties of their single “parts” studied in isolation from each other.

Cybernetics too challenged the “mechanistic” conception that the universe was based on the “operation of anonymous particles at random” and emphasized “the search for new approaches, for new and more comprehensive concepts, and for methods capable of dealing with the large wholes of organisms and personalities”. (von Bertalanffy 1972a, 28)

If applied in a nothing-but sense, a further reductionistic bias would be that cybernetics apparently does not distinguish between “dead and living process”, a distinction considered irrelevant in cybernetic considerations (Günther 1957, 25). Cybernetics looks for universal laws: when it was defined as “the science of control and communication in the animal and the machine” (Wiener 1948a), this “definition indicated (a) that a state of in-control depends upon a flow of information, and (b) that the laws governing control are universal, i.e. do not depend on the classical dichotomy between organic and inorganic systems” (von Foerster 1974, 2).

### 5.2. Ideology and world view

Bertalanffy’s program is tightly connected to specific humanistic values (cf. Pouvreau 2013a, 911–913, 2014a, 213). He claimed that the old, scientifically grounded, mechanistic world view brought much miseries to humankind (Pouvreau and Drack 2007; Pouvreau 2013a, 715–720). This world view should be replaced by a holistic one in line with a science of organization, wholeness and dynamics. It still had to be developed in order to be in position to fulfil this mission:

Last century’s mechanistic world picture was closely related to the domination of the machine, the theoretical view of living beings as machines and the mechanization of man himself. Concepts, however, which are coined by modern scientific developments, have their most obvious exemplification in life itself. Thus, there is a hope that the new world concept of science is an expression of the development toward a new stage in human culture. (von Bertalanffy 1969a, 259)

According to Bertalanffy, cybernetics harbours a problematic world view.^48^ He noted a “certain danger of the concept of homeostasis”, which implies a machine model of the organism with feedback in a pre-arranged structure. von Bertalanffy (1956b, 17) feared that overemphasis on homeostasis would have far-reaching implications: “the so-called well-adjusted individual will be the ultimate goal of education; that is, a well-oiled robot maintaining itself in optimal biological, psychological, and social homeostasis”.

[T]he primary organic regulations [of an organism] do not fit into any machine-conception in both its aspects, namely, structural conditions for the order of processes, and one-way causality. (von Bertalanffy 1951a, 355)

Thus, homeostasis together with an equilibrium conception might – besides science – also mislead the world view because “a great deal of biological and human behavior is beyond the principles of utility, homeostasis and stimulus-response” (von Bertalanffy 1962, 14). The concept of homeostasis would be useless for explaining non-utilitarian behaviour, which is quite common in humans. According to Bertalanffy’s “philosophical anthropology” (see Pouvreau 2013a, 270–288, 2014a, 197, 209), humans, as “symbolic animals”, behave in ways that cannot be reduced to homeostasis and utilitarian behaviour:

We may also say that homeostasis is inappropriate as an explanatory principle for those human activities that are non-utilitarian, that is, not serving the primary needs of self-preservation and survival and their secondary derivatives, as is the case with many cultural manifestations. (Bertalanffy 1965b, 1102–1103)

Although the homeostasis model transcends older mechanistic schemes by acknowledging directiveness in self-regulating circular processes, it still adheres to the machine theory of the organism. This also applies to a second aspect. An essential component of the mechanistic view is a utilitarian conception which is deeply rooted in the economic outlook of the 19th and early twentieth century. […] The utilitarian or economic viewpoint also prevails in the concept of homeostasis: The organism is essentially envisaged as an aggregate of maintenance mechanisms. However, there seem to be plenty of non-utilitarian structures and functions in the living world. (von Bertalanffy 1959, 275)

The main inconvenience that general systemologists felt about cybernetics is rooted in the machine view applied to humans or other living organisms. Of course, stating that “the human brain behaves very much like the machines” (Wiener 1948b) and calling the animals “living machines” (Wiener 1948a, xv) gave rise to Bertalanffy’s objection:

The student in “systems science” receives a technical training which makes systems theory – originally intended to overcome current overspecialization – into another of the hundreds of academic specialties. Moreover, systems science, centered in computer technology, cybernetics, automation and systems engineering, appears to make the systems idea another – and indeed the ultimate – technique to shape man and society ever more into the “megamachine” which Mumford […] has so impressively described in its advance through history. (von Bertalanffy 1969a, vii-viii)

Bertalanffy tried to safeguard “systems thinking” from being linked to gloomy predictions of the future. This caused him to contrast the intentions or implications of general systemology with those of cybernetics and others. The title and content of one of his papers, “General Systems Theory and a new view of the nature of man” (von Bertalanffy 1968), well reflect his attitude. He knew that all great human ideas – and thus also those about systems – lend themselves to inhuman purposes (von Bertalanffy 1971, 90; Pouvreau 2014a, 213–214) and he strongly expressed his sorrow by warning that “system” was becoming a mere catchword that nonetheless fostered severe political evolutions:

Moreover, the dangers of “systems” are apparent. Systems designers, analysts, researchers, behavioral engineers, and members of similar professions – New Utopians, as Boguslaw […] called them – contribute to or even lord over the industrial-military establishment. Elaborating weapons systems, dominating advertising, mass media, and propaganda, and in general preparing a cybernetic society of the future, they must of necessity tend to exclude or suppress the “human element.” For this human element, individualistic, capricious, often unforeseeable, is precisely the unreliable part of the “megamachine” (to use L. Mumford’s term) of the present and future; hence, it should be either replaced by computers and other hardware or else made “reliable” – that is, as machinelike, automated, uniform, conformist, and controlled as possible. “Systems” thus appears to be the epitome of the automated wasteland of *Brave New World* and *1984*. (Bertalanffy 1969b, 33–34; cf. also 1969a, 10)

Bertalanffy considered cybernetics and GST (in the “narrow sense”) as belonging, respectively, to the “mechanistic” and the “organismic” trends of systems science (von Bertalanffy 1967, 64). He maintained that the world view behind cybernetics is a machine picture of the organism, whereas GST’s is organismic:

The first [mechanistic trend] is understandably connected with technological developments such as control theory, cybernetics, system analysis in commerce and industry, and so forth. A systems (that is, multivariable) approach is obviously imperative to deal with the complex problems in modern society; it carries the danger of making the human individual ever more into a small wheel of the social “megamachine.” (von Bertalanffy 1965b, 1111)

However, Wiener made a great effort to develop a humanistic view. In particular, he warned against the automata “related social dangers” (Wiener 1948a, vii) which can arise when the machine is only used for profits and worshiped as a new brazen calf rather than increasing man’s leisure and enriching the spiritual life (Wiener 1954). He especially emphasized the problem of competition as it pertains to the potential practical outcomes of ever more sophisticated machines:

I have said that this new development has unbounded possibilities for good and for evil. For one thing, it makes the metaphorical dominance of the machines, as imagined by Samuel Butler, a most immediate and non-metaphorical problem. It gives the human race a new and most effective collection of mechanical slaves to perform its labor. Such mechanical labor has most of the economic properties of slave labor, although, unlike slave labor, it does not involve the direct demoralizing effects of human cruelty. However, any labor that accepts the conditions of competition with slave labor accepts the conditions of slave labor, and is essentially slave labor. The key word of this statement is competition. It may very well be a good thing for humanity to have the machine remove from it the need of mental and disagreeable tasks, or it may not. I do not know. (Wiener 1948a, 27)

Wiener’s answer to these problems was “to have a society based on human values”, and he was critical about considering mere market mechanisms as a universal norm of societal organization. He was aware of the potential use of cybernetics for good and for evil. This prompted his suggestion to make the broader public understand the trend and the bearing of their work, and “to confine our personal efforts to those fields, such as physiology and psychology, most remote from war and exploitation” (Wiener 1948a, 28).

## 6. Cybernetics and its rapprochement to the project of general systemology

It would be mistaken to interpret the project of “general systemology” and “cybersciences”^49^ as different but essentially related manifestations of a single research movement, at least from the point of view of their origins and their constitution. For one, as opposed to “cybersciences”, general systemology as originally conceived by Bertalanffy as well as Boulding and Rapoport after him, was not intended to be connected to war purposes. It was in their minds intimately related to definite ethical commitments (pacifism in particular) and philosophical questionings. Its genesis was mainly rooted in theoretical problems having almost no connection with technology: most concerned biology, psychology and social sciences. Moreover, “cybersciences” cannot even be regarded as a homogeneous set (Pouvreau 2013a, 765–805). The central issue here is to understand why and how all those lines of research came into contact, dialogued and, to a certain extent, associated themselves.

This confluence is grounded in certain fundamental characters that “cybersciences” shared with the Bertalanffian project and with most of Rapoport’s, Boulding’s, Gerard’s and Miller’s works prior to 1954: (1) an awareness of the necessity to develop new strategies in order to grasp the problems resisting the “meristic” epistemologies and their classical “mechanistic” declensions; (2) the subsequent development of systemic concepts and principles; (3) a will to inscribe this development in the mathematical sphere as far as possible and, overall, to overcome the confinement of holistic categories to metaphysics or to the realm of intuition; (4) the general character – that is, abstract and transcending established disciplinary subdivisions – of the concepts and principles in question; (5) an effort to overcome the old dualism between “natural science” and “humanities”^50^ by means of those constructions; (6) an interdisciplinary communication and cooperation regarded as constitutive of the undertaken research; and (7) a deliberate questioning of the established structure of scientific disciplines and the simultaneous ambition to enrich each of these disciplines^51^ (Pouvreau 2013a, 807).

Nonetheless, these common traits are in themselves not sufficient to account for the confluence of those lines of research that differ significantly from the point of view of their respective problems and of the “system” concepts that each of them developed (Pouvreau 2013a, 670–808). Rather, the explanation must be sought in the particularly uncertain status of “cybersciences” at least until the mid-1950s. Their concept of “theory” indeed oscillated between three meanings and therefore remained undetermined (cf. also: Müller 1996, 158–181). The first meaning was classical, positivist in its inspiration and modelled on the physical sciences: theory as a set of logically connected statements that are empirically both grounded and interpretable. From this standpoint, the remarkable dilemma was that the universalism of their constructs depended on a mathematical sophistication which in turn imposed very restrictive conditions on the possibility of their respective applications; this put the “cybersciences” in a position to solve only very particular problems or to provide concurring models for a single concrete systemic behaviour, without any possibility to decide between those models. The second meaning of their concept of “theory” was that of a heuristic, aimed at guiding empirical researches. But this was also a problem: the application of “cyberscientific” constructions, particularly in the social realm, always presupposed that the issue being studied could be grasped with the very specific fundamental terms in which those constructions were formulated: no discussion was undertaken about the relevance of this presupposition, let alone any systematic clarification – except, to a certain extent, in the works of the founders of “operations research”. Third, the concept of “theory” also had a praxeological meaning: it was meant as a constructive interpretation aimed at studying the spectrum of the possibilities of action and at rationalizing the latter. This also entailed the problematic opposition between the specificity of the norms of rationality and of the ideals of action (controllability, optimization, etc.) that were imposed by the “cyberscientific” models.

The systemologists, particularly Bertalanffy from the 1930s onwards, were specifically setting up a project which, from the onset, made extensive use of those three meanings of the concept of “theory”. At the same time, they strived to lay the philosophical foundation that would enable their consistent coexistence and overcoming the aforementioned apparent contradictions: at least since the early 1950s, all the concerned issues had been discussed with more or less depth in their initial contributions to “philosophical systemology” related to perspectivism, philosophy of systems science, ontology, logic, methodology, axiology and praxeology. The further deepening of these discussions has in fact been a major characteristic of the whole development of the systemological project, especially in Rapoport’s work (Pouvreau 2013a, 333–420, 739–746, 864–914, 2014a, 197–210).

The “cybersciences” framework lacked any clarification of the function of theory and of the concept of scientific law. This was a problem in that these new sciences introduced a radical novelty which was theorized some years later by Herbert A. Simon^52^: according to him, the “science of systems” only truly takes its meaning as the “science of the artificial”, that is, of artifacts – machines or social organizations – and of theoretical models. The difficulty here was the profound challenge to scientific ideals and philosophy. The universalist ideal of science was thus challenged insofar as the problems solved by “cybersciences” usually depended on historical contingencies: the state of technology and of social organization of work. The distinctions between discovery, justification and application contexts were completely muddled by those sciences, and one could qualify the “cybersciences” neither as basic nor as applied sciences. They fused basic research and advanced technology but were not oriented toward specific problems: they typically presented theoretical generalizations of certain problematical situations characterized by teleological categories. “Cybersciences” tended in that way to challenge the ideal of basic science as disinterested research into the truth about “matter of facts”. They were deeply involved in two processes that profoundly affected the ideals of autonomy and objectivity: the “scientifization” of technology and the politicization of basic science. These new sciences had been and still were associated with war purposes. This impacted their concepts and methods, fuelling a growing skepticism with regard to the social functions of science. It also cast doubts on the idea that science, although an end in itself, should be only pragmatically judged with regard to its technical and social usefulness, and even with regard to its dangers (Pouvreau 2013a, 806–808).

Without trying to remedy it before the 1950s, some “cyberscientists” were aware of this set of uncertainties regarding epistemology as well as the general philosophy of science and of its social function.^53^ This clearly played an essential role in their rapprochement with the founders of the SGSR (Bertalanffy, Rapoport, Boulding, Gerard). The views of the latter formed a wide and sufficiently open alternative, philosophically as well as scientifically, to enable the general “systemological” project towards which the “cyberscientists” converged to play the role of an organizing pole of this scientific society in which constructive discussions seemed likely to overcome the shortcomings of all involved parts, while equally enabling something like a “self-awareness” of a “system movement” to emerge. Moreover, there was clearly a common institutional interest: since the involved researchers (the proponents of general systemology as well as the cyberscientists) mostly worked outside the established disciplinary frameworks, their conjunction also held out the promise to favour the formation of new and more relevant frameworks. This included university departments or scientific centres completely devoted to the development of their research and to teaching their results (Pouvreau 2013a, 806–808).

## 7. Conclusion

Overcoming specialization and integrating various scientific fields was a common aim of Bertalanffy and cyberneticists, even though they started from different standpoints. First order cybernetics was largely concerned with engineering. Here, very useful theoretical models and methods were developed that turned out to be applicable in different fields in a relatively straight forward way. In contrast, Bertalanffy’s “systemological” project was from the onset very significantly concerned with philosophy in general (especially theory of knowledge, metaphysics and axiology) as well as with ideological issues. And it was chiefly elaborated with regard to the fields of biology and psychology. The criticism towards an alleged “realism” of general systemology cannot be upheld: it must be strictly rejected with regard to the trained philosopher Bertalanffy and almost all other systemologists. Constructivism stemming from second order cybernetics and Bertalanffy’s perspectivism are not equal, but they are also not mutually exclusive. This provides an opportunity for common future efforts. On scientific grounds, cybernetics starts with a machine view of different kinds of systems (technical devices, organisms, brains, social patterns), whereas systemology starts with an organismic view of various systems (organisms, biocenoses, psyche and cultures). Bertalanffy emphasized the (gradual) difference between regulation in an open system and in a homeostatic feedback system. He pointed out the problem of trying to apply the feedback mechanism to research issues where it is not applicable; arguing that other types of explanation had to be sought. He also emphasized the connections of these different approaches to different world views, and the fact (also acknowledged by Rosenblueth and Wiener [1945] concerning cybernetics) that the underlying models, insofar as their scopes are necessarily limited, must therefore be used cautiously. This was Bertalanffy’s primary critique against cybernetics: he warned against the world view that might arise from a mere feedback and homeostasis thinking. He felt that forgetting the model character of the cybernetic constructs was a great danger with regard to the view of humans and their societies. Bertalanffy also logically warned against the potential perversions that may come from his own research program. His underlying idea (clearly moulded to a significant extent by his own tragic experience with Nazism, discussed in Pouvreau [2009]) was that some models may be more dangerous than others, depending on the context of their genesis and reception: some can lend themselves more easily than others to hypostasis of concepts.

Bertalanffy had a very general view of systemology as a program and hermeneutics for the systemic interpretation of the “real” (Pouvreau 2013a; 2014a). This systemology implies, according to him, a new orientation, a new paradigm in contrast to the “blind laws of nature” in classical science (von Bertalanffy 1972b, 27). Cybernetics also contrasts with classical science by putting circularity and teleology on its agenda. The view of “general systemology” as instigated by Bertalanffy and developed by him and other “systemologists” such as Klir was however that *cybernetics is “but” a major contribution to the implementation of “general systemology”*. This contribution mainly concerns “basic theoretical systemology”, but also to various extents “applied theoretical systemology”, “systems technology” and “philosophical systemology”. We call Bertalanffy’s more specific contribution *“organismic systemology”* (thus mostly referring to his so-called “GST in the narrow sense”) and call the cyberneticists’ contribution *“cybernetic systemology”*. Both would therefore be *different but complementary aspects of the development of “general systemology”* as a general science of the systemic interpretation of the “real” (the so-called “GST in the wide sense”). We suggest the term “general systemology” for the common endeavour. Suggestions such as “systems science” as an umbrella term (Heylighen and Joslyn 2002) are problematic because they have already been used with another meaning: von Bertalanffy (1962, 3) understood “systems science” as the fields of GST concerned with systems theorizing (i.e. both “applied” and “basic” theoretical systemologies, in Pouvreau’s terminology). Moreover, it should be seriously taken into account that Bertalanffy explicitly involved many *philosophical* issues and fields in the elaboration and implementation of his project. “General systemology” (or perhaps only “systemology”) as an umbrella term is more neutral. Remaining fully faithful to Bertalanffy’s inspiration, the concept to which it refers may in particular help avoiding reductionism in “systems research”.

## Notes on contributors


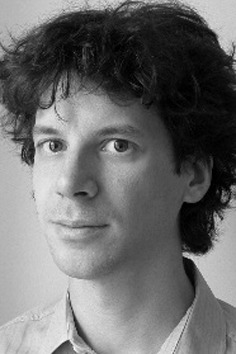
Manfred Drack is Austrian. He was trained as a mechanical engineer and studied biology. After conducting research at the Center for Biomimetics at the University of Reading, he received a doctorate from the University of Vienna with a thesis on biomimetics and eco-design. He worked in several projects at the Center for Appropriate Technology of the Vienna University of Technology. Rupert Riedl brought him back to the University of Vienna. After one year in Olaf Wolkenhauer’s group at the University of Rostock, he was working at the Department of Theoretical Biology, University of Vienna. Now he is at the University of Tübingen.


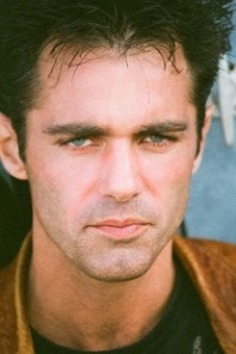
David Pouvreau is French. He is a postgraduate in mathematics (agrégation) and holds a doctorate in the history and philosophy of science (School of Advanced Studies in Social Sciences, Paris). He is a fellow at the Bertalanffy Center for the Study of Systems Science in Vienna and is associate researcher at the Department of Philosophy of the University of Toulouse II. He is continuing his research in the history and philosophy of “general system theory”, which was the subject of his doctoral studies. He has published several papers on the subject, as well as a biography of Ludwig von Bertalanffy.

## Notes

1. Detailed reasons for using this term are provided in Pouvreau (2014a, 172ff).

2. The issues raised here are also interesting for recent developments in systems biology. In this field, the feedback concept and aspects from control engineering play an important role, but little attention is paid to Bertalanffy’s ideas.

3. Many of the concepts used by Luhmann (e.g. 1984) stem from developments in (second order) cybernetics (e.g. autopoiesis, self-reference, self-organization, operational closure, structural coupling, observing and re-entering) with references not only to Humberto Maturana, Francisco Varela and Heinz von Foerster but also to George Spencer Brown.

4. Glanville refers to: “As a preliminary comment, cybernetics is obviously the dynamic complement of systemics. This means that it implicitly introduces the time dimension in many models that would otherwise be merely structural and static” (François 2006, 88). Glanville puts forward his argument in a section entitled “Cybernetics and General Systems Theory” and then changes François’ “systemics” to “systems”.

5. The term information is apparently not meant in a Shannon and Weaver sense. Later cyberneticists preferred the term “organizationally closed” instead of “information-tight” (Scott 2004, 1369).

6. Ashby’s machine concept refers to a “state determined system”. More details are provided in the subsection on Ashby.

7. Feedback is, more or less clearly, connected to several other concepts: interdependence, mutual causality, circular causality, recursion, self-reference, knowledge of results and causal loops. “Some limit the concept of feedback to homeostasis and control, implicitly or explicitly restricting it to refer to negative feedback. Some derive the dynamic behavior of a system from its feedback structure, while others fail to connect feedback to dynamics. Still others try to draw dynamic inferences from feedback structure but produce demonstrably false conclusions” (Richardson 1991, 3–4).

8. This holds for both first and second order cybernetics. The current subsection, however, is confined to the former.

9. One can also conceive alternative mechanisms for picking up a pencil in which no feedback via visual sensing is involved. Somebody could look at a pencil, then close his/her eyes and pick up the pencil without watching. This involves memory and probably other processes but definitely no visual feedback. Wiener (1948a, 7) also pointed to the possibility of kinesthetic or proprioceptive processes being involved. This might be correct or not. Note, however, that searching solely for feedback mechanisms constraints the scope of possible explanations.

10. Some confusion arises because the authors also state that “[a]ll purposeful behavior may be considered to require negative feed-back” (Rosenblueth et al. 1943, 19) This contradicts the dichotomy in the scheme in which there is also non-feedback purposeful behaviour.

11. Wisdom (1951, 4) distinguished three types of feedback, according to the origin of energy. The regulating energy and mechanism can then be termed “simple feedback” with energy from the main power, “amplification feedback” with energy from the local source, or “mixed feedback” where both energy sources are employed.

12. The “richness of connection” and the intention that systems can show more or less “wholeness” (Ashby 1956, 65, 68) is reminiscent of Bertalanffy’s “progressive segregation”, where all parts of a system are initially interconnected but with time certain connections are lost and several areas act independently of one another.

13. The concept of “higher order” statistics refers to an approach where phenomena at a higher hierarchical order are investigated without reducing them to phenomena or concepts at lower levels. This is similar to Boltzmann’s statistical approach in thermodynamics where the connections between macro level variables, such as temperature and pressure, are investigated without reduction to the molecular or atom level (cf. Pouvreau and Drack 2007, 307–309).

14. Cf. Heims (1991) and http://www.asc-cybernetics.org/foundations/history2.htm Accessed 14 March 2014.

15. Deutsch published in 1949 (partly reprinted in 1968) an outline of a “cybernetic model of man and society” using “the advantages of the use of electric nets, nervous systems and societies as analogues of each other” in order to interpret by means of the cybernetic concepts of retroaction, message and network the theories of learning, values, consciousness, will, autonomy, integrity and freedom, as well as the problem of social cohesiveness.

16. This is, of course, not new, and van Glasersfeld (1995, 149) refers to the theories of relativity and quantum mechanics, where the problem of the observer and the observed arises, as the “most powerful and encouraging corroboration of the cybernetician’s disengagement from the dogma of objectivity”. Note that Bohr’s and Heisenberg’s doctrine of complementarity, which has had an early and far-reaching influence on Bertalanffy’s “perspectivist” theory of knowledge (Pouvreau 2013a, 295–597 in particular), had earlier emphasized the necessity of such a disengagement, at least with regard to the *classical* separation between subject and object as a necessary condition of objectivity (Heisenberg 1931; Bohr 1934).

17. A similar meta-theoretical character is found in the “basic theoretical systemology” subfield of “general systemology” (Pouvreau 2014a, 195, 215–216).

18. So termed by Glasersfeld (e.g. 1984).

19. Vico is well known for his *verum et factum convertuntur*-principle, which translates to “the true and the made are […] convertible”, or in another expression *verum esse ipsum factum*: “the true is precisely what is made”. “Vico emphasizes that science should be conceived as the ‘genus or mode by which a thing is made’” (Costelloe 2012). For this reason, history is more certain than natural sciences. In a printed discussion, Bertalanffy also refers to Vico: “The idea that we can only ‘understand’ things when we can ‘make’ them, is, I believe, first found in Nicholas of Cusa and later, of course, in Giambattista Vico. In a very real sense, we create the world around us. For this very reason, knowledge is never ‘truth’ but a ‘perspective’ of reality.” (Koestler and Smithies 1969, 152) In this regard, Bertalanffy was influenced by Anderle (1956). With regard to Vico’s “verum factum” thesis, see also Arendt (1998).

20. “They [natural philosophers, physicists] learned that reason only perceives that which it produces after its own design; that it must not be content to follow, as it were, in the leading-strings of nature, but must proceed in advance with principles of judgment according to unvarying laws, and compel nature to reply its questions.” (Kant [1787] 2003, B XIII, translation taken from http://www.gutenberg.org/cache/epub/4280/pg4280.txt).

21. Piaget’s “genetic” epistemology was informed by his psychological studies of the development of intelligence; this constructivist epistemology aimed at explaining the progressive emergence of knowledge structures: see Pouvreau and Drack (2007, 295). Cassirer (1927) and von Bertalanffy (1928) saw Nicholas of Cusa as the father of a critical, anti-relativistic constructivism (cf. Pouvreau 2013a, 276).

22. Jakob von Uexküll used the feedback concept in his *Umwelt* theory. The experience of the individual is generated by the coordination of its “receptor” and “effector” organs in a functional circle (*Funktionskreis*). This circle also includes what he called a *Merkwelt* and a *Wirkwelt* (von Uexküll 1973, 158 in particular).

23. Lorenz interpreted Kant’s *a priori* as *a posteriori* of evolution (Lorenz 1941).

24. Mario Bunge was and remains a rare exception (cf. Bunge 1979).

25. The term “meristic” [*meristisch*] was coined in reference to the Greek term *μερος* (“part”) as an antonym to the term “holistic” (Anderle 1960, 149).

26. Feedback was missing in the simple stimulus-response scheme (Watson’s behaviorism), to which Bertalanffy here alluded.

27. This point is relevant with regard to the connection between open systems theory and thermodynamics of irreversible processes.

28. Bertalanffy’s “open system theory” and “generalized kinetics” are other parts of this “basic theoretical systemology”.

29. The probability that Ashby discovered Bertalanffy’s works through Wisdom’s paper is very high, because: (1) von Bertalanffy (1950), Wisdom (1951) and Ashby (1952a) were published in the same journal; (2) Wisdom (1951, 6–12) dedicated a long part of his criticism of cybernetics to Ashby’s works, and it is hard to imagine that the latter ignored this paper since he referred to Wisdom in Ashby (1952b); (3) Ashby, in (1952a), refers to von Bertalanffy (1950).

30. The feedback concept that Wiener refers to stems from control engineering (Wiener 1948a, 6–7).

31. As a matter of fact, there are several definitions, according to the considered structures (algebraic, topological, etc.).

32. For details on the construction of the homeostat together with drawings and pictures, see Ashby (1960, 100ff).

33. That is, the variables intimately related to the “survival” of the system. They are connected in such a way that the change of one of them significantly affects all the others (Ashby 1952b, 41–43).

34. “The basic rule for adaptation by trial and error is: If the trial is unsuccessful, change the way of behaving; when and only when it is successful, retain the way of behaving […] It is equivalent to the following formulation: (1) When the essential variables are not all within their normal limits (i.e. when the trial has failed), *no* state of the system *S* is to be equilibrial (for the rule here is that *S* must go to some other state). (2) When the essential variables *are* all within normal limits then *every* state of *S* is to be equilibrial (i.e. *S* is to be in neutral equilibrium)” (Ashby 1960, 84).

35. “The presence of step-mechanisms in a state-determined system enables both schools to be right, provided that those who maintain the determination are speaking of the system which comprises *all* the variables, while those who maintain the possibility of spontaneous change are speaking of the main variables only. For the whole system, which includes step-mechanisms, has one field only, and is completely state-determined. But the system of main variables may show as many different forms of behavior as the step-mechanisms possesses combinations of value. And if the step-mechanisms are not accessible to observation, the change of the main variables from one form of behavior to another will seem to be spontaneous, for no change or state in the main variables can be assigned as its cause.” (Ashby 1960, 95).

36. A simple example given by Ashby (1956, 127) was the following one: the variety in the human sexes is 1 bit; if a school only admits boys, the variety of sexes in the school is zero. The difference in variety expresses a “constraint”.

37. More precisely the tenth theorem, which states that if noise appears in a message, the amount of noise that can be eliminated by a correction channel is limited by the amount of information which can be carried through this channel (Shannon and Weaver 1949, 68).

38. This interpretation has been adopted by Atlan (1979, 42–43).

39. Cf. Cavallo (1979, 108). The mathematical theory of dynamic systems as used by Lotka, Volterra and Bertalanffy seems to entail statements which deserve the status of “general system laws” as well, with regard to a constructivist point of view rejecting the idea that the laws “freely run in nature” (a Bertalanffian expression).

40. “If the system is to be in some sense ‘self-organizing’, the ‘self’ must be enlarged to include [a] variable α [of which] the change comes from some outside agent, acting on the system as input […] Thus the appearance of being ‘self-organizing’ can be given only by [a] machine coupled to another machine” (Ashby 1962, 266–269 in particular).

41. Cf. also Pouvreau (2014a, 202–203).

42. Cybernetics has never convincingly shown how it could tackle such issues. To be fair, Bertalanffy's organismic systemology also mainly failed in that regard (points (1) and (4) are exceptions, notably in view of his models of animal growth); but it defined an original problematic, raised stimulating questions and initiated a theoretical program.

43. The (M,R)-system of Rosen (1958) is according to von Bertalanffy et al. (1977, 145–146; this is a posthumous second edition of Bertalanffy [1953a], which was extended by Beier and Laue) a transition from the general relational systems of Rashevsky to biological regulation and bio-cybernetics. The theory of (M,R)-systems is characterized as a theory of biological regulation on a relational level. Cybernetics, in this regard, appears as a very specialized, though important, theory which only captures one class of systems that show self-regulation (von Bertalanffy et al. 1977, 145–146).

44. They stated that the theory they present could also be termed “general cybernetics” (Mesarovic and Takahara 1975, 2).

45. The relationship between “internal” and “external” (black box), “structural” and “functional”, as well as continuous and discrete modes of description was discussed in Pouvreau (2013a, 876–878, 2014a, 202).

46. This definition was discussed in Pouvreau (2013a, 52–53). See also Pouvreau and Drack (2007, 286).

47. Interesting in this context are Rosen’s (1979, 183) considerations: “Now the essence of feedback control is that it is *error*-*actuated*; in other words, the stimulus to corrective action is the discrepancy between the systems’s actual present state and the state the system should be in. Stated otherwise, a feedback control system must already be departing from its nominal behavior before control begins to be exercised. On the other hand, we know from introspection that many, if not most of our own conscious activities are generated in quite a different way. We typically decide what to do *now* in terms of what we perceive will be the effects of our action at some *later* time. To take a simple example: if we are walking in the woods, and see a bear appear from behind a tree, we need not wait until he is upon us before vacating the area. Rather we leave immediately, because we can *anticipate* the unpleasant consequences of continuing our walk. The vehicle by which we anticipate is in fact a *model*, which enables us to pull the future into the present. The stimulus for our action is in fact not simply the sight of the bear; it is the prediction or output of our model under these conditions. This simple example contains the essence of anticipatory behavior. It involves the concept of *feedforward*, rather than feedback. […] In a feedback system, as we have seen, control is error-actuated, and serves to correct a system performance which is already deteriorating. In a feedforward system, on the other hand, system behavior is preset, according to some model relating present inputs to their predicted outcomes. […] Despite the ubiquity of feedforward modes of control, at all biological levels, anticipatory behavior of this kind has hardly begun to be seriously studied”.

48. Cf. Pouvreau (2014a, 230).

49. “Cybersciences” also include operations research. The term was coined by Evelyn Fox Keller (1994, 304–305).

50. Including psychology.

51. There are some overlappings with the seven parallels pointed out by Bertalanffy, as mentioned above.

52. “[A]rtifacts are not apart from nature. They have no dispensation to ignore or violate natural law. At the same time they are adapted to human goals and purposes. They are what they are in order to satisfy our desire to fly or to eat well. As our aims change, so too do our artifacts – and vice versa”. “If science is to encompass these objects [artifacts] and phenomena in which human purpose as well as natural law are embodied, it must have means for relating these two disparate components” (Simon 1969, 3).

53. Considerations of Wiener (1948a, vii, 27) related above can illustrate this fact. Ackoff (1960, 7–8, 1963, 1974, 11–17) also had such reflections in connection with the problem of the status of “operations research”, but some years later.

## References

[CIT0001] Ackoff R. L. (1960). Systems, Organizations and Interdisciplinary Research. *General Systems: Yearbook of the Society for General Systems Research*.

[CIT0002] Ackoff R. L. (1963). General System Theory and Systems Research: Contrasting Conceptions of Systems Science. *General Systems: Yearbook of the Society for General Systems Research*.

[CIT0003] Ackoff R. L. (1974). The Systems Revolution. *Long Range Planning*.

[CIT0004] Anderle O. F. (1956). Giambattista Vico als Vorläufer einer morphologischen Geschichtsbetrachtung [Giambattista Vico as Forerunner of a Morphological Way of Viewing History]. *Die Welt als Geschichte*.

[CIT0005] Anderle O. F. (1960). Die Monadologie G.W. Leibniz’ – Modellfall einer allgemeinen Ganzheitstheorie in nuce [The Monadology of G.W. Leibniz – A Model Case for a General Theory of Wholeness in a Nutshell]. *Zeitschrift für Ganzheitsforschung*.

[CIT0006] Arendt H. (1998). *The Human Condition*.

[CIT0007] Ashby W. R. (1940). Adaptiveness and Equilibrium. *Journal of Mental Science*.

[CIT0008] Ashby W. R. (1952a). Can a Mechanical Chessplayer Outplay its Designer?. *The British Journal for the Philosophy of Science*.

[CIT0009] Ashby W. R. (1952b). *Design for a Brain*.

[CIT0010] Ashby W. R. ([1955] 1956). Review of Biophysik des Fliessgleichgewicht by Ludwig von Bertalanffy [Review of Ludwig von Bertalanffy’s Biophysics of the Flux Equilibrium]. *The British Journal for the Philosophy of Science*.

[CIT0011] Ashby W. R. (1956). *An Introduction to Cybernetics*.

[CIT0012] Ashby W. R. (1958). General Systems Theory as a New Discipline. *General Systems: Yearbook of the Society for General Systems Research*.

[CIT0013] Ashby W. R. (1960). *Design for a Brain*.

[CIT0014] Ashby W. R., von Foerster H., Zopf G. W. (1962). Principles of the Self-Organizing System. *Principles of Self-organization*.

[CIT0015] Atlan H. (1979). *Entre le cristal et la fumée – Essai sur l’organisation du vivant* [Between Crystal and Smoke – Essay on the Organization of Life].

[CIT0016] Bavink B. (1949). *Ergebnisse und Probleme der Naturwissenschaften* [Results and Problems of the Natural Sciences].

[CIT0017] von Bertalanffy L. (1928). *Nikolaus von Kues* [Nicolas Cusanus].

[CIT0018] von Bertalanffy L. (1929a). Vorschlag zweier sehr allgemeiner biologischer Gesetze. Studien über theoretische Biologie III [Suggestion of Two General Biological Laws. Studies in Theoretical Biology III]. *Biologisches Zentralblatt*.

[CIT0019] von Bertalanffy L. (1929b). Die Teleologie des Lebens. Eine kritische Erörterung [The Teleology of Life. A Critical Analysis]. *Biologia Generalis*.

[CIT0020] von Bertalanffy L. (1937). *Das Gefüge des Lebens* [The Structure of Life].

[CIT0021] von Bertalanffy L. (1949a). Zu einer allgemeinen Systemlehre [Towards a General Systemology]. *Biologia Generalis*.

[CIT0022] von Bertalanffy L. (1949b). *Das biologische Weltbild – Die Stellung des Lebens in Natur und Wissenschaft* [The Biological World View – The Position of Life in Nature and Science].

[CIT0023] von Bertalanffy L. (1950). An Outline of General System Theory. *The British Journal for the Philosophy of Science*.

[CIT0024] von Bertalanffy L. (1951a). General System Theory: A New Approach to Unity of Science – 6. Toward a Physical Theory of Organic Teleology – Feedback and Dynamics. *Human Biology*.

[CIT0025] von Bertalanffy L. (1951b). Theoretical Models in Biology and Psychology. *Journal of Personality*.

[CIT0026] von Bertalanffy L. (1953a). *Biophysik des Fließgleichgewicht – Einführung in die Physik offener Systeme und ihre Anwendung in der Biologie* [Biophysics of the Flux Equilibrium – Introduction into the Physics of Open Systems and its Application in Biology].

[CIT0027] von Bertalanffy L. (1953b). Philosophy of Science in Scientific Education. *Scientific Monthly*.

[CIT0028] von Bertalanffy L. (1955a). General Systems Theory. *Main Currents in Modern Thought*.

[CIT0029] von Bertalanffy L. (1955b). An Essay on the Relativity of Categories. *Philosophy of Science*.

[CIT0030] von Bertalanffy L. (1956a). *Die Beiträge der Systemtheorie zum Wissenschaftsdenken der Gegenwart* [The Contributions of System Theory to Present Scientific Thinking].

[CIT0031] von Bertalanffy L. (1956b). Some Considerations on Growth in its Physical and Mental Aspects. *Merrill-Palmer Quarterly*.

[CIT0032] von Bertalanffy L., McChandler Brooks C., Cranefield P. F. (1959). Modern Concepts on Biological Adaptation. *The Historical Development of Physiological Thought*.

[CIT0033] von Bertalanffy L. (1962). General System Theory – A Critical Review. *General Systems: Yearbook of the Society for General Systems Research*.

[CIT0034] von Bertalanffy L. (1965a). Zur Geschichte theoretischer Modelle in der Biologie [Towards the History of Theoretical Models in Biology]. *Studium Generale*.

[CIT0035] von Bertalanffy L., Arieti S. (1965b). General System Theory and Psychiatry. *American Handbook of Psychiatry*.

[CIT0036] von Bertalanffy L. (1967). *Robots, Men and Minds*.

[CIT0037] von Bertalanffy L. (1968). General Systems Theory and a New View of the Nature of Man.

[CIT0038] von Bertalanffy L. (1969a). *General System Theory – Foundations, Development, Applications*.

[CIT0039] von Bertalanffy L., Gray W., Duhl F. J., Rizzo N. D. (1969b). General Systems Theory and Psychiatry – An Overview. *General Systems Theory and Psychiatry*.

[CIT0040] von Bertalanffy L., Lohman M. (1970). Biologie und Weltbild [Biology and World View]. *Wohin führt die Biologie?*.

[CIT0041] von Bertalanffy L., Kurzrock R. (1971). Vorläufer und Begründer der Systemtheorie [Forerunners and Founders of System Theory]. *Systemtheorie: Forschung und Information*.

[CIT0042] von Bertalanffy L., Klir G. (1972a). The History and Status of General System Theory. *Trends in General Systems Theory*.

[CIT0043] von Bertalanffy L., Kurzrock R. (1972b). Vorläufer und Begründer der Systemtheorie [Forerunners and Founders of System Theory]. *Systemtheorie*.

[CIT0044] von Bertalanffy L., Beier W., Laue R. (1977). *Biophysik des Fließgleichgewichts* [Biophysics of the Flux Equilibrium].

[CIT0045] Bohr N. (1934). *Atomic Theory and the Description of Nature*.

[CIT0046] Bunge M. (1979). *Treatise on Basic Philosophy. Vol. 4 – Ontology II: A World of Systems*.

[CIT0047] Cannon W. B. (1932). *The Wisdom of the Body*.

[CIT0048] Cassirer E. (1910). *Substanzbegriff und Funktionsbegriff. Untersuchungen über die Grundfragen der Erkenntniskritik* [Concept of Substance and Concept of Function. Studies on the Fundamental Issues of the Critique of Knowledge].

[CIT0049] Cassirer E. (1927). *Individuum und Kosmos in der Philosophie der Renaissance* [Individual and Cosmos in Renaissance Philosophy].

[CIT0050] Cassirer E. (1929). *Philosophie der symbolischen Formen. Dritter Teil: Phänomenologie der Erkenntnis* [Philosophy of the Symbolic Forms. Part Three: Phenomenology of Knowledge].

[CIT0051] Cavallo R. E. (1979). Systems Research Movement: Characteristics, Accomplishments, and Current Developments. *General Systems Bulletin*.

[CIT0052] Costelloe T., Zalta E. N. (2012). Giambattista Vico. *The Stanford Encyclopedia of Philosophy*.

[CIT0053] Deutsch K. W., Buckley W. (1968). Toward a Cybernetic Model of Man and Society. *Modern Systems Research for the Behavioral Scientist*.

[CIT0054] Dubrovsky V. (2004). Toward System Principles: General System Theory and the Alternative Approach. *Systems Research and Behavioral Science*.

[CIT0055] von Foerster H., Preiser F. E. (1973). On Constructing a Reality. *Environmental Design Research*.

[CIT0056] von Foerster H. (1974). *Cybernetics of Cybernetics*.

[CIT0057] François C., Trappl R. (2006). The Observer Reobserved. *Cybernetics and Systems – Proceedings of the 18th European Meeting on Cybernetics and Systems Research*.

[CIT0058] Gergely T. (1979). On the Unity Between Cybernetics and General System Theory. *Kybernetes*.

[CIT0059] Glanville R., Arnold D. P. (2014). Cybernetics: Thinking Through the Technology. *Traditions of Systems Theory: Major Figures and Contemporary Developments*.

[CIT0060] von Glasersfeld E., Watzlawick P. (1984). An Introduction to Radical Constructivism. *The Invented Reality – How Do We Know What We Believe We Know?*.

[CIT0061] von Glasersfeld E. (1995). *Radical Constructivism*.

[CIT0062] Goldsmith M. H., Reckmeyer W. J. (1981). The Concept of Teleology and its Development in System Theory and Cybernetics. *General Systems Research and Design: Precursors and Future, Proceedings of the 25th Annual North American Meeting of the Society for General Systems Research, Toronto, Canada, January 6–9*.

[CIT0063] Gray W., Reckmeyer W. J. (1981). Frontiers of General Systems and Cybernetics Research: Humanistic Considerations. *General Systems Research and Design: Precursors and Future, Proceedings of the 25th Annual North American Meeting of the Society for General Systems Research, Toronto, Canada, January 6–9*.

[CIT0064] Günther G. (1957). *Das Bewußtsein der Maschinen – Eine Metaphysik der Kybernetik* [The Consciousness of Machines – A Metaphysic of Cybernetics].

[CIT0065] Hammond D. (2003). *The Science of Synthesis*.

[CIT0066] Heims S. J. (1991). *The Cybernetics Group*.

[CIT0067] Heisenberg W. (1931). Kausalgesetz und Quantenmechanik [Causal Law and Quantum Mechanics]. *Erkenntnis*.

[CIT0068] Heylighen F., Joslyn C. (2001). Second-Order Cybernetics.

[CIT0069] Heylighen F., Joslyn C., Meyers R. A. (2002). Cybernetics and Second-order Cybernetics. *Encyclopedia of Physical Science and Technology*.

[CIT0070] Howland D. (1963). Cybernetics and General Systems Theory. *General Systems: Yearbook of the Society for General Systems Research*.

[CIT0071] Kant I. ([1787] 2003). *Kritik der reinen Vernunft* [Critique of Pure Reason].

[CIT0072] Keller E. F. (1994). The Body of a New Machine: Situating the Organism Between Telegraphs and Computers. *Perspectives on Science*.

[CIT0073] Klir G., Rose J. (1970). On the Relation Between Cybernetics and General Systems Theory. *Progress in Cybernetics*.

[CIT0074] Klir G. (1978). *Applied General Systems Research: Recent Developments and Trends*.

[CIT0075] Klir G. (1988). Correspondence Systems Profile. *Systems Research*.

[CIT0076] Klir G. (2001). *Facets of Systems Science*.

[CIT0077] Koestler A., Smithies J. R. (1969). *Beyond Reductionism – New Perspectives in the Life Sciences*.

[CIT0078] Köhler W. (1955). Direction of Processes in Living Systems. *The Scientific Monthly*.

[CIT0079] Locker A. (1998). The Present Status of General System Theory, 25 Years after Ludwig von Bertalanffy’s Decease – A Critical Overview.

[CIT0080] Lorenz K. (1941). Kants Lehre vom Apriorischen im Lichte gegenwärtiger Biologie [Kant’s Doctrine of the *a priori* in the Light of Contemporary Biology]. *Blätter für deutsche Philosophie*.

[CIT0081] Lotka A. J. (1925). *Elements of Physical Biology*.

[CIT0082] Luhmann N. (1984). *Soziale Systeme* [Social Systems].

[CIT0083] Luhmann N. (2002). *Einführung in die Systemtheorie* [Introduction to System Theory].

[CIT0084] McCool J. P. (1980). Cybernetics and General Systems – A Unitary Science?. *Kybernetes*.

[CIT0085] McCulloch W. S., Pitts W. (1943). A Logical Calculus of the Ideas Immanent in Nervous Activity. *The Bulletin of Mathematical Biophysics*.

[CIT0086] Mead M., Foerster H. v., White J. D., Peterson L. J., Russell J. K. (1968). Cybernetics of Cybernetics. *Purposive Systems – Proceedings of the First Annual Symposium of the American Society for Cybernetics*.

[CIT0087] Mesarovic M. D., Takahara Y. (1975). *General Systems Theory: Mathematical Foundations*.

[CIT0088] Mesarovic M. D., Takahara Y. (1989). *Abstract Systems Theory*.

[CIT0089] Miller J. G. (1978). *Living Systems*.

[CIT0090] Mittasch A. (1938). Bemerkungen über Anstoß- und Erhaltungskausalität in der Natur [Comments on Impulse Causality and Conservation Causality in Nature]. *Die Naturwissenschaften*.

[CIT0091] Müller K. (1996). *Allgemeine Systemtheorie* [General System Theory].

[CIT0092] Nagel E. (1961). *The Structure of Science: Problems in the Logic of Scientific Explanation*.

[CIT0093] Pouvreau D. (2009). *The Dialectical Tragedy of the Concept of Wholeness – Ludwig von Bertalanffy’s Biography Revisited*.

[CIT0094] Pouvreau D. (2013a). Une histoire de la ‘systémologie générale’ de Ludwig von Bertalanffy – Généalogie, genèse, actualisation et postérité d’un projet herméneutique [A History of Ludwig von Bertalanffy’s General Systemology – Genealogy, Genesis, Implementation and Posterity of a Hermeneutical Project].

[CIT0095] Pouvreau D. (2013b). The Project of ‘General Systemology’ Instigated by Ludwig von Bertalanffy: Genealogy, Genesis, Reception and Advancement. *Kybernetes*.

[CIT0096] Pouvreau D. (2014a). On the History of Ludwig von Bertalanffy’s ‘General Systemology’, and on its Relationship to Cybernetics – Part II: Contexts and Developments of the Systemological Hermeneutics Instigated by von Bertalanffy. *International Journal of General Systems*.

[CIT0097] Pouvreau D., Arnold D. P. (2014b). The Hermeneutical System of General Systemology: Bertalanffian and Other Early Contributions to its Foundations and Development. *Traditions of Systems Theory: Major Figures and Developments*.

[CIT0098] Pouvreau D., Drack M. (2007). On the History of Ludwig von Bertalanffy’s ‘General Systemology’, and on Its Relationship to Cybernetics – Part I: Elements on the Origins and Genesis of Ludwig von Bertalanffy’s ‘General Systemology’. *International Journal of General Systems*.

[CIT0099] Prigogine I., Stengers I. (1979). *La nouvelle alliance* [Order out of Chaos].

[CIT0100] Rapoport A., Klir G. J. (1972). The Uses of Mathematical Isomorphism in GST. *Trends in General Systems Theory*.

[CIT0101] Richardson G. P. (1991). *Feedback Thought in Social Science and Systems Theory*.

[CIT0102] Rosen R. (1958). A Relational Theory of Biological Systems. *The Bulletin of Mathematical Biophysics*.

[CIT0103] Rosen R. (1969). Putting a Science Back on the Track – Review of L. von Bertalanffy’s General System Theory. *Science*.

[CIT0104] Rosen R. (1979). Old Trends and New Trends in General Systems Research. *International Journal of General Systems*.

[CIT0105] Rosenblueth A., Wiener N. (1945). The Role of Models in Science. *Philosophy of Science*.

[CIT0106] Rosenblueth A., Wiener N. (1950). Purposeful and Non-purposeful Behavior. *Philosophy of Science*.

[CIT0107] Rosenblueth A., Wiener N., Bigelow J. (1943). Behavior, Purpose and Teleology. *Philosophy of Science*.

[CIT0108] Ruyer R. (1954). *La cybernétique et l’origine de l’information*.

[CIT0109] Scott B. (2004). Second‐order Cybernetics: An Historical Introduction. *Kybernetes*.

[CIT0110] Shannon C. E., Weaver W. (1949). *The Mathematical Theory of Communication*.

[CIT0111] Simon H. A. (1969). *The Sciences of the Artificial*.

[CIT0112] Taylor R. (1950). Comments on a Mechanistic Conception of Purposefulness. *Philosophy of Science*.

[CIT0113] Tibaldeo R. F. (2008). Beyond Ontological Reductionism, and Towards an Ontological Revolution – A Comparison between Norbert Wiener’s Cybernetics, Ludwig von Bertalanffy’s General System Theory, and Hans Jonas.

[CIT0114] von Uexküll J. (1973). *Theoretische Biologie* [Theoretical Biology].

[CIT0115] Umpleby S. A. (1990). The Science of Cybernetics and the Cybernetics of Science. *Cybernetics and Systems*.

[CIT0116] Umpleby S. A., Dent E. (1999). The Origins and Purposes of Several Traditions in Systems Theory and Cyberneics. *Cybernetics and Systems*.

[CIT0117] Ungerer E. (1966). *Die Wissenschaft vom Leben – Eine Geschichte der Biologie, Band III: Der Wandel der Problemlage der Biologie in den letzten Jahrzehnten* [The Science of Life – A History of Biology, Volume 3: The Transformation of the Problems of Biology During the Last Decades].

[CIT0118] Wiener N. (1948a). *Cybernetics*.

[CIT0119] Wiener N. (1948b). Cybernetics. *Scientific American*.

[CIT0120] Wiener N. (1954). *The Human Use of Human Beings – Cybernetics and Society*.

[CIT0121] Wisdom J. O. (1951). The Hypothesis of Cybernetics. *The British Journal for the Philosophy of Science*.

[CIT0122] Wittezaele J. J., Garcia T. (1992). *À la recherche de l’école de Palo Alto* [In Search of the Palo Alto School].

[CIT0123] Young O. R. (1964). A Survey of General Systems Theory. *General Systems: Yearbook of the Society for General Systems Research*.

